# BCL7B, a SWI/SNF complex subunit, orchestrates cancer immunity and stemness

**DOI:** 10.1186/s12885-023-11321-3

**Published:** 2023-08-30

**Authors:** Sayaka Higuchi, Yuji Suehiro, Luna Izuhara, Sawako Yoshina, Akira Hirasawa, Shohei Mitani

**Affiliations:** 1 Institute for Comprehensive Medical Sciences, Tokyo Women’s Medical University, Tokyo, 162-8666 Japan; 2 Department of Physiology, Tokyo Women’s Medical University School of Medicine, Tokyo, 162-8666 Japan; 3 Department of Genomic Drug Discovery Science, Graduate School of Pharmaceutical Sciences, Kyoto University, 46-29 Yoshida-Shimo-Adachi-Cho, Sakyo-Ku, Kyoto, 606-8501 Japan

**Keywords:** BCL7B, SWI/SNF complex, H3K27me3, Recognition centroid sequence changes, Immune system, Cancer stem cells

## Abstract

Cancer is one of the main causes of human death. Here, we focus on the B-cell lymphoma 7 protein family member B (BCL7B) gene, an accessory subunit of the SWI/SNF chromatin-remodelling complex. To characterize the function of BCL7B, heterozygous BCL7B-deficient stomach cancer cell lines were generated with the CRISPR/Cas9 genome editing system. The comprehensive gene expression patterns were compared between parental cells and each ΔBCL7B cell line by RNA-seq. The results showed marked downregulation of immune-related genes and upregulation of stemness-related genes in the ΔBCL7B cell lines. Moreover, by ChIP-seq analysis with H3K27me3 antibody, the changes of epigenetic modification sequences were compared between parental cells and each ΔBCL7B cell line. After machine learning, we detected the centroid sequence changes, which exerted an impact on antigen presentation. The regulation of BCL7B expression in cancer cells gives rise to cancer stem cell-like characteristics and the acquisition of an immune evasion phenotype.

**Supplementary Information:**

The online version contains supplementary material available at 10.1186/s12885-023-11321-3.

## Introduction

Cancer is one of the main causes of human death. To overcome the disease, we first need to understand the underlying pathological mechanisms. For example, the concept of cancer stem cells has been widely accepted by cancer researchers [1–3]. This theory suggests that cancer stem cells survive after treatment and become initiators of cancer recurrence. Recurring cancers undergo a deleterious evolution to acquire drug resistance and genetic diversity [4, 5]. However, the precise mechanisms by which cancer stem cells are generated and maintained among tumour cells are still unclear. In this study, to better understand cancer pathology, we focused on the B-cell lymphoma 7 protein family member B gene (BCL7B), which is deleted in Williams-Beuren syndrome, a rare neurodevelopmental disorder [6]. Recently, BCL7B deficiency was suggested to confer a risk of several malignancies, such as haematologic cancer in patients with Williams–Beuren syndrome [7, 8]. In addition, it has been reported that in the *bcl-7* mutant of *Caenorhabditis elegans*, the nuclei in stem cells, which are called seam cells in this organism, and the nuclei in the human stomach cancer cell line (the Kato III cell line) are enlarged when BCL7B expression is downregulated by siRNA [9]. Notably, BCL7B siRNA-treated Kato III cells showed malignant characteristics [9]. However, BCL7B function in the pathology of Williams–Beuren syndrome and in the course of malignant progression of cancers remains unclear. Recently, BCL7B was reported to be an accessory molecule in the SWI/SNF complex and associated mainly with BRG1-associated factors (BAFs) [10–12]. The SWI/SNF complex, which consists of approximately 15 subunits, is a representative chromatin-remodelling machine [12, 13]. Moreover, different combinations of subunits in this complex have been suggested to result in different cellular functions. SWI/SNF subunit combinations are known at least two forms, BAF and polybromo-associated BAF (PBAF) complexes [12, 14]. Chromatin remodelling is a very important mechanism for normal organism development and cell differentiation [15], and abnormality in this process has been suggested to be involved in carcinogenesis [16, 17]. In fact, certain subunit molecules have been reported to be tumour suppressor genes [14, 16, 17]. In this study, to characterize the function of BCL7B and thus gain a better understanding of cancer pathology, BCL7B-deficient cancer cell lines (ΔBCL7B-1, ΔBCL7B-2 and ΔBCL7B-3 cell lines) were generated with the CRISPR/Cas9 genome-editing system. The comprehensive gene expression patterns of the mutant lines were compared with those of the parent cell line by RNA-seq. We thus reveal the cell characteristics of the ΔBCL7B cells and describe BCL7B function in detail, generating hints for overcoming cancer pathology.

## Results

### Establishment of heterozygous BCL7B-deficient Kato III cell lines

**Fig. 1:**
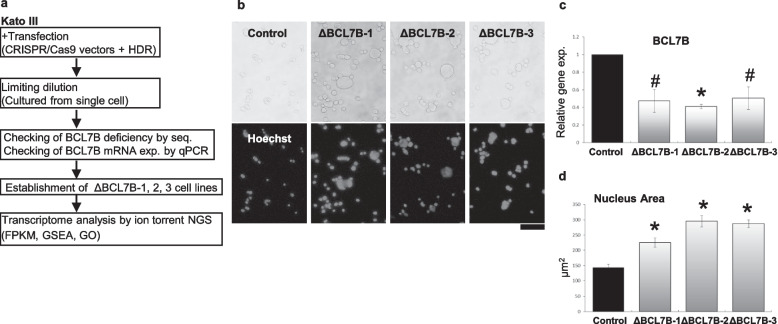
Establishment of BCL7B-deficient cell lines. **a** Schematic representation of the experimental design. **b** Images showing the phase contrast (top) and Hoechst 33,342 fluorescence (bottom) of cultured BCL7-parent (control) and mutant (ΔBCL7B-1–3) Kato III cells. Scale bar, 100 μm. **c** The expression levels of the BCL7B gene was determined by quantitative real-time PCR (qPCR) and is presented relative to control cells. SE (*n* = 3). The symbols show the statistical significance (*p < 0.005, #*p* < 0.05). **d** The nuclear area was stained by Hoechst 33,342 and measured by ImageJ (https://imagej.nih.gov/ij/index.html). Error bars, SEs (Control Kato III; *n* = 173, ΔBCL7B-1; *n* = 354, ΔBCL7B-2; n = 266; ΔBCL7B-3; *n* = 490). The asterisks show statistical significance (*p < 0.005)

The BCL7B gene in *Homo sapiens* encodes three isoforms, with each carrying a common transcription initiation sequence. We designed two gRNAs for use in CRISPR/Cas9 on the basis of the common initiation sequence, and we established three haploid BCL7B-deficient cell lines (BCL7BΔ1-3 cell lines), which showed significant downregulation of BCL7B expression (Fig. 1c). The details of the cell-establishment process are provided in the Materials and Methods. (Figure 1a, Extended Data Fig. 1 and Materials and Methods). We stained the cells with Hoechst 33,342 and measured the nuclear area (Fig. 1b). The nuclear sizes between Kato III cells (control) and BCL7B-deficient Kato III cells (ΔBCL7B-1, ΔBCL7B-2, and ΔBCL7B-3 cells) were compared. The results showed that the nuclei in the BCL7B-deficient cells were significantly larger than those in the control cells (Fig. 1d), consistent with a previous work performed with BCL7B siRNA [9]. We also tried to establish homozygous BCL7B-deficient cell lines; however, we were unsuccessful.

### RNA-seq analysis indicated that immunity-related GO terms were enriched with genes that were downregulated in the BCL7B-deficient cell lines

**Fig. 2:**
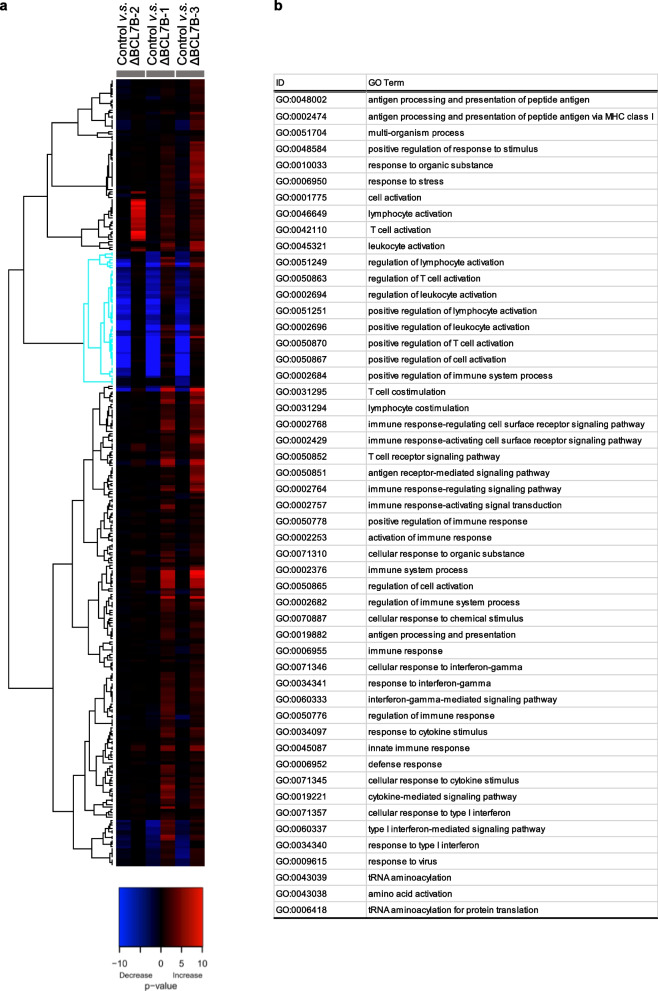
Heatmap based on RNA-seq analysis of BCL7B-deficient cells and control Kato III cells. **a** Gene set enrichment analysis (GSEA). The expression of the majority of genes in the blue cluster was downregulated. Each sample is listed in two lines: the left line indicates downregulated genes, and the right line indicates upregulated genes. **b** Gene Ontology (GO) terms in the blue cluster shown in (**a**). The most enriched GO terms were related to immunity

To understand the function of BCL7B, the mRNA expression levels were compared between control Kato III cells and BCL7B-deficient cell lines by RNA-seq. Then, we performed gene set enrichment analysis (GSEA) to identify Gene Ontology (GO) terms associated with BCL7B deficiency (Fig. 2). We found a downregulated cluster, which is indicated by blue lines, in comparison with control cells, with other clusters showing variable expression patterns, depending on the cell line (Fig. 2a). We noticed that the almost all of GO terms in the downregulated cluster are related to immunology, such as “GO:0048002 antigen processing and presentation of peptide antigen” (Fig. 2b).

### RNA-seq analysis of BCL7B-deficient cells indicated the marked downregulation of antigen presentation-related genes

**Fig. 3:**
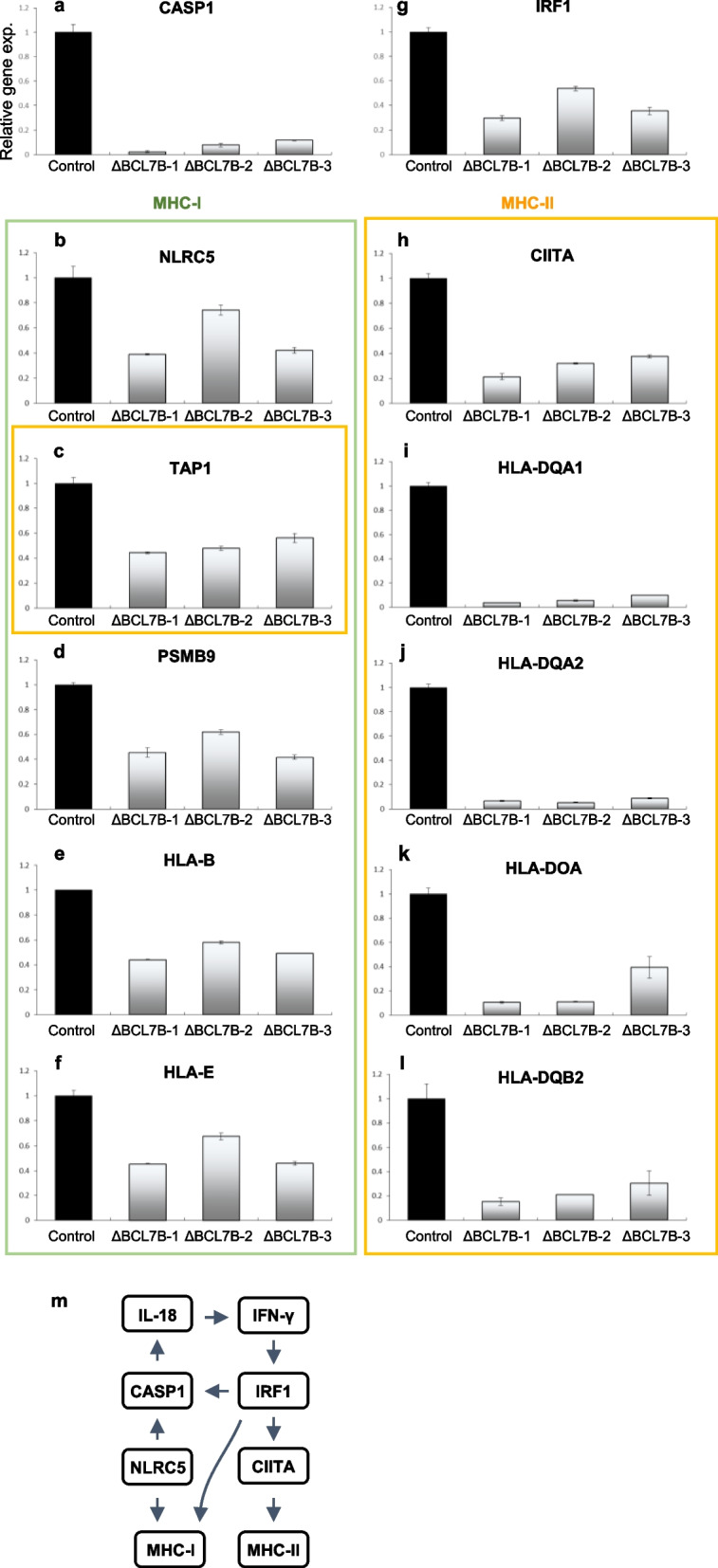
The marked downregulation of antigen presentation genes found via a RNA-seq analysis of BCL7B-deficient cells is reported as the FPKM. **a** The expression levels of the CASP1 gene, an initiator of inflammation and the immune response cascade, in terms of FPKM. **b** The expression levels of the NLRC5 (CITA) gene, a key cotransactivator of the MHC class I pathway. **c**-**f** The expression levels of representative NLRC5-downstream genes: (**c**) TAP1, (**d**) PSMB9 (LMP2), (**e**) HLA-B, and (**f**) HLA-E. **g** The expression levels of the IRF1 gene, a transcription factor of CIITA, which has also been implicated in regulating class I MHC expression. **h** The expression levels of the CIITA gene, a key transactivator of the MHC class II pathway. **c**, **i**-**l** The expression levels of representative CIITA-downstream genes: (**c**) TAP1, (**i**) HLA-DQA1, (**j**) HLA-DQA2, (**k**) HLA-DOA, and **(l**) HLA-DQB2. **m** Simplified diagram showing the relationships of antigen presentation molecules

The expression levels of mRNAs are represented by FPKM (fragments per kilobase of exon per million reads mapped), which was calculated on the basis of data obtained with an Ion Torrent next-generation sequencing (NGS) machine. We used the values obtained for the BCL7B-deficient cells to estimate changes in individual gene expression compared to their expression in control cells. The downregulated mRNAs in all three BCL7B-deficient cell lines are listed in Extended Data Table 1. To identify downregulated genes, molecules with expression levels associated with an FPKM higher than 10 in control cells were analysed. Interestingly, we noticed that many antigen presentation-related genes, such as HLA-B, HLA-E, HLA-DQA1, HLA-DQA2, HLA-DOA, and HLA-DQB2, were on the list generated with these data. Notably, we realized that the expression of NLRC5 (CITA) and CIITA, which are key transcription factors related to antigen presentation genes, was downregulated. NLRC5 is an MHC class I transactivator [18, 19], and CIITA is an MHC class II molecule transactivator [20]. Moreover, the expression of interferon regulatory factor 1 (IRF1), which is a transcription factor of CIITA [21] and has been implicated in the regulation of MHC class I molecule expression, was also downregulated [19]. Additionally, the expression of CASP1, which initiates inflammation and the immune response cascade [22], was also downregulated in the BCL7B-deficient cell lines. The FPKM ratios between the three mutant cell lines and the control cells for antigen presentation-related genes are shown in Fig. 3a-l. To show the regulatory relationship among these genes, a diagram of the known pathways is depicted in Fig. 3m [18–21, 23]. We confirmed the downregulation of antigen presentation related genes, NLRC5, CIITA, etc., in BCL7B-deficient cells, by real-time PCR (Extended data Fig. 2). The HLA-related genes were not suitable to perform qPCR analysis, because the construction of gene-specific primers was difficult due to the very similar sequences of HLA family genes. The results support the findings of the GO analysis (Fig. 2). We also analysed another cancer cell line to confirm the BCL7B functions we identified. The U937 cell line showed favourable characteristics, including a relative high level of BCL7B expression and relatively lower expression levels for other BCL7 family genes, e.g., BCL7A/C, similar to Kato III cells (Extended Data Fig. 3a). We generated three heterozygous BCL7B-deficient U937 cell lines (UΔBCL7B-1, UΔBCL7B-2, and UΔBCL7B-3 cell lines) with significant downregulation of BCL7B expression (Extended Data Fig. 3b and c). The BCL7B-deficient U937 cells were characterized by a large cell size and a tendency towards lower expression of NLRC5 and CIITA (Extended data Fig. 3, d, e, f and g), similar to the characteristics of the BCL7B-deficient Kato III cells. This result suggests that BCL7B function is commonly involved in antigen presentation through the expression of NLRC5 and CIITA.

### The downregulation of antigen presentation-related genes is implicated as a malignancy indicator

Recently, the immune evasion of tumor cells was recognized as an intrinsic property of quiescent stem cells in vivo, resulting from systematic downregulation of genes encoding the antigen presentation machinery, including MHC class I molecule genes [24]. We speculated that the transition to lower expression levels of antigen presentation-related genes might be related to cancer malignancy due to their relationship to the cancer stem cell-like state transition. To examine this hypothesis, we compared our experimental data with publicly available gene expression profiles, namely the profile of chronic leukaemia (K562) cells were compared to acute leukaemia cells (HL-60, MOLT-4, CCRF-CEM, SR, and RPMI-8226 cells), and Kato III cells were compared to BCL7B-deficient Kato III cells. We used a dataset (GDS4296) of leukaemia cell line gene expression, which was obtained through the NCBI website, as the comparison groups. We performed a GSEA to identify GO terms enriched with genes involved in cancer malignancy. The results showed that there was a commonly downregulated cluster, which is indicated with blue, of majority genes in comparison with K562, chronic leukemia and KatoIII cells (Extended Data Fig. 4a). We noticed that the almost all of GO terms in the downregulated cluster were related to immunology, such as GO:0002376 immune system process (Extended Data Fig. 4b).

**Fig. 4:**
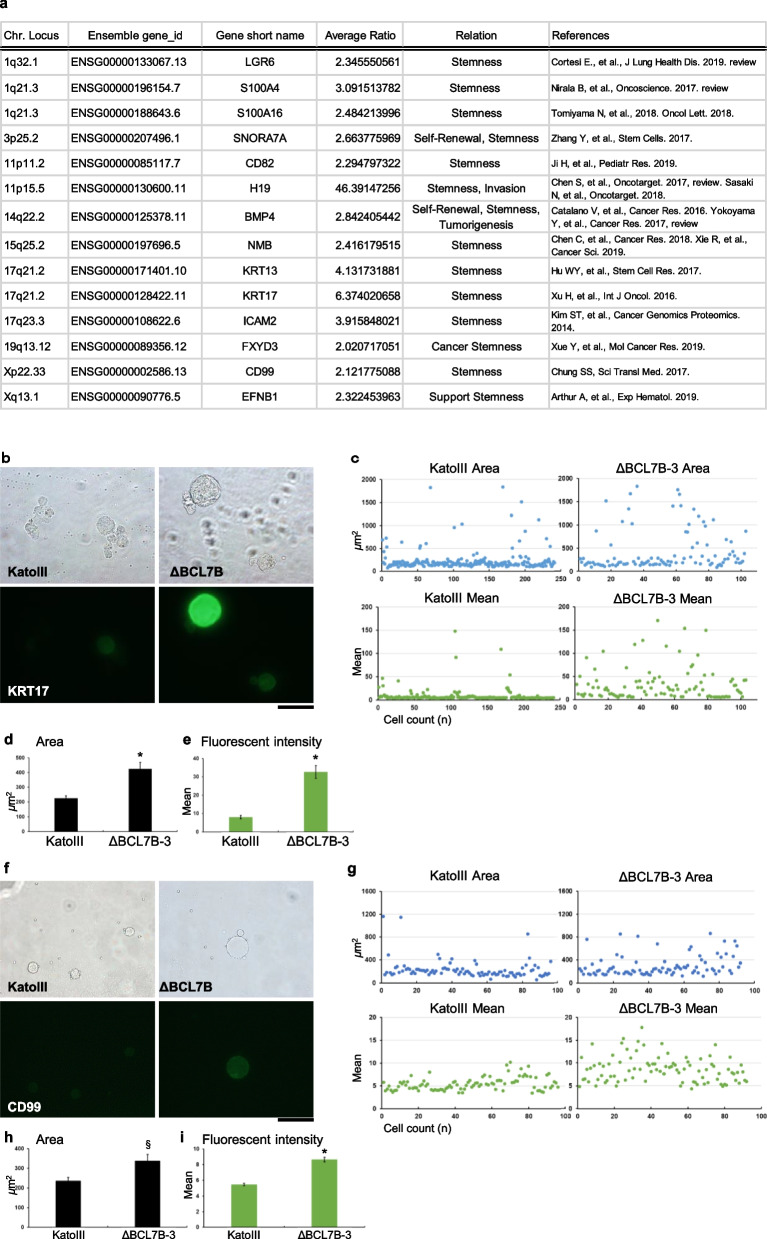
The expression of cancer stemness-related genes in BCL7B-deficient cell lines. **a** List of cancer stemness-related genes in Extended Data Table 3. **b** Phase contrast (top) and KRT17-stained images (bottom) of KatoIII and ΔBCL7B KatoIII cells. Scale bar, 50 μm. **c** Cell size and KRT17 fluorescence intensity per area were measured by ImageJ. **d** Graph showing a cell size comparison. **e** Graph comparing the fluorescence levels of KRT17. Error bars, SEs (control KatoIII cells, *n* = 241, ΔBCL7B cells, *n* = 103). The asterisks show statistical significance (*p < 0.005). **f** Phase contrast (top) and CD99-stained images (bottom) of KatoIII and ΔBCL7B KatoIII cells. Scale bar, 50 μm. **g** Cell size and CD99 fluorescence intensity per area were measured by ImageJ. **h** Graph showing cell size comparisons. **i** Graph comparing the fluorescence level of CD99. Error bars, SEs (control KatoIII cells, *n* = 96, ΔBCL7B, *n* = 92). The asterisk and symbol show the statistical significance (*p < 0.005, §*p* < 0.01)

### BCL7B-deficient cells transitioned into malignant cells

Next, we noticed that some microRNAs (miRNAs) were unexpectedly included in the downregulated gene list, *e.g*., the Let-7 miRNA family, MIRLET7A1, MIRLET7A3, MIRLET7D and MIRLET7F1 miRNAs, as shown in Extended Data Table 2a. The downregulation of Let-7 miRNAs is known as a malignancy indicator [25, 26]. To search for miRNA targets of MIRLET7A, MIRLET7D and MIRLET7F, we used an online resource, miRDB (http://www.mirdb.org/), for miRNA target prediction and functional annotation [27]. The predicted targets (score ≥ 95) of MIRLET7A, MIRLET7D and MIRLET7F are shown in Extended Data Table 2b and c (b, 3-prime site; c, 5-prime site). Interestingly, many nuclear localization proteins, which are shown in the purple columns in the lists, were identified as targets, suggesting a causal relationship with these miRNAs and nuclear abnormalities in BCL7B-deficient cells. Additionally, the mRNAs with expression upregulated by twofold or more in all three BCL7B-deficient cell lines are listed in Extended Data Table 3 and Supplementary Table 1. We found that the BCL7B-deficient cell lines showed a higher expression of numerous malignancy markers, which are shown in the light red columns in the lists (Supplementary Table 1) and included BMP4 [28, 29], S100A4 [30], S100A16 [31], RAB38 [32], Wnt11 [33], LGR6 [34], CD82 [35], and H19 [36]. Importantly, higher expression of LGR6 (Chr.1q), S100A4 (Chr.1q), S100A16 (Chr.1q), SNORA7A (Chr.3p), CD82 (Chr.11p), H19 (Chr.11p), BMP4 (Chr.14q), NMB (Chr.15q), KRT13 (Chr.17q), KRT17 (Chr.17q), ICAM2 (Chr.17q), FXYD3 (Chr.19q) or CD99 (Chr. Xp and Chr. Yp) has been suggested to be indicative of a cancer stem cell property (Fig. 4; references are in the Supplementary information), indicating a causal relationship with stemness in BCL7B-deficient cells. Then, we measured stemness marker expression levels. Specifically, we measured the level of KRT17, a marker of proliferation, invasion, and poor prognosis in cancer, as a stemness marker [37]. The results showed that the BCL7B-deficient cell lines exhibited higher expression of KRT17 (Fig. 4). We then measured the expression of another stem cell marker, CD99, which is a marker of acute myeloid leukaemia (AML) and myelodysplastic syndrome (MDS) stem cells [38]. The results showed that the BCL7B-deficient cell lines exhibited higher expression of CD99 (Fig. 4). These results support the findings of the RNA-seq analysis (Supplementary Table 1).

### Localization changes of MES-2, an H3K27me3 methyltransferase, in bcl-7 RNAi C. elegans

Next, we were interested in the mechanism by which the expression of antigen presentation-related genes is changed in BCL7B-deficient cells. BCL7B is an SWI/SNF complex accessory molecule. The SWI/SNF complex, a chromatin-remodelling complex, is assumed to exhibit several functions, such as epigenetic regulation, nucleosome mobilization, nucleosome ejection, and histone dimer exchange [39]. We focused on changes in epigenetic regulation in BCL7B-deficient cells. We first examined the relationship between *bcl-7* knockdown and epigenetic changes in *C. elegans*. The results of our inquiry indicated that the localization of only MES-2::GFP was markedly changed (Extended Data Fig. 5), although the localizations of other epigenetic factors did not change. The MES-2 protein, a subunit of PRC2-like complex, is a H3K27-specific methyltransferase in *C. elegans *[40].

**Fig. 5:**
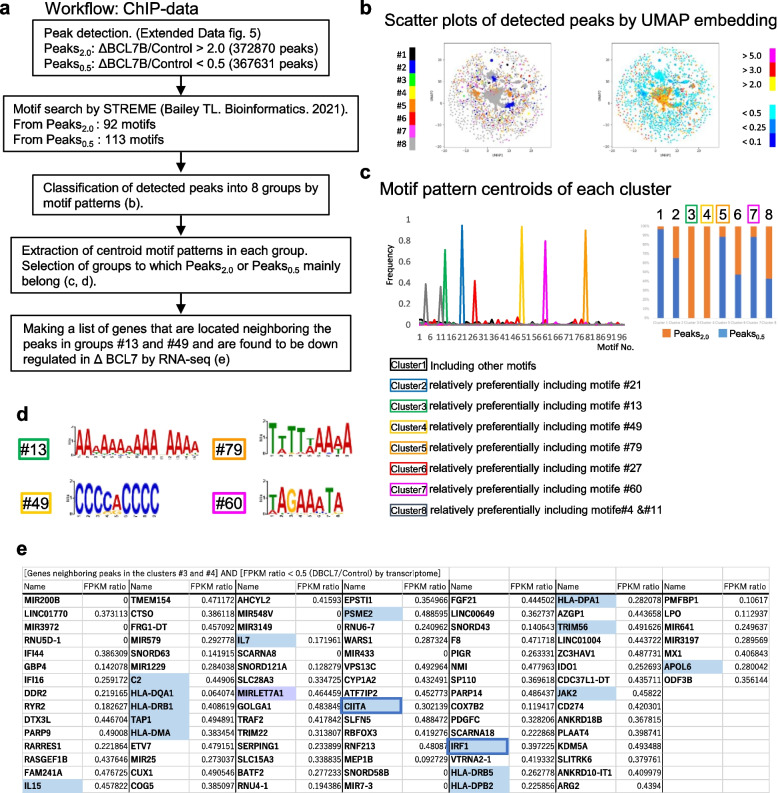
Detection of regulated sequences in ΔBCL7B cells identified by machine learning. **a** The ChIP-seq data analysis workflow. **b**-**d** From ChIP-seq data of the control parent cell line and ΔBCL7B cell lines, 372,870 regions (Peaks2.0) and 367,631 regions (Peaks0.5) were identified as peaks preferentially obtained in the ΔBCL7B group and control group, respectively. **b** All the converted peak information is displayed as scatter plots after UMAP embedding. The colour coding is based on the clusters (left panel) or the ratios of read depth of the ΔBCL7B group to the control group (right panel). **c** Cluster centroids clusters are shown in a line chart (upper left pane). The horizontal axis indicates the index of short motifs, and the vertical axis indicates the frequency of each motif. The centroids of Clusters 3 and 4, that consisted only of peaks derived from the ΔBCL7B group, indicate high frequency of short motifs #13 and #49, respectively. The Clusters 5 and 7 preferentially consisted of peaks derived from the control group. The centroids indicate high frequencies of short motifs #79 and #60. **d** The sequence logos of short motifs #13 and #49 (left panels) and #79 and #60 (right panels) are shown. **e** The RNA-seq data indicated that the genes downregulated in the ΔBCL7B group and located within 10 kb of the peaks belonged to Clusters 3 and 4 and are shown in tables. Immune response-related genes (blue) and the MIRLET7A gene (purple) were identified

### Localization changes of EZH2, a MES-2 homologs gene in human, in BCL7B-deficient cells

Next, we tested immunohistochemically to detect the localization change of EZH2, a MES-2 homologous gene in humans, in BCL7B-deficient cells. Then, we observed the variety of localization in KatoIII control cells, and the localization of EZH2 were categorized into four types, Half to Whole (HW), Edge (E), Partial (P) and Dark (D) (Extended data Fig. 6a). Localization patterns were compared between KatoIII and BCL7B deficient cells. The results showed that region of EZH2 localization tended to expand in BCL7B deficient cells (Extended data Fig. 6b).

### The machine-learning detection of gene sequences regulated by H3K27me3 in BCL7B-deficient KatoIII cells

The histone mark H3K27me3 is known as a gene-silencing modificaiton [41]. Therefore, using an H3K27me3 antibody, we performed ChIP-seq analysis to evaluate the epigenetic changes between the control cells and the BCL7B-deficient cell lines. We expected to find changes in the regulated sequences with epigenetic marks. The library for ChIP-seq was prepared according to the Ion Torrent protocols, and ChIP-seq was performed on an Ion Torrent Ion Chef/Ion Proton system. The workflow showing ChIP-seq data analysis performed for the detection of regulated sequences is presented in Fig. 5a. First, by compared to control cells, the sequence regions with H3K27me3 marks in the ΔBCL7B group were detected for each peak type; 372,870 high peak regions (Peaks_2.0) and 367,631 low peak regions (Peaks_0.5) were identified (Extended Data Fig. 7). The nucleotide sequences of these peaks were then analysed, and short motifs were identified. These short motifs were further used to convert each peak into a numerical vector representing motif frequency. Ultimately, the vectors were used in machine learning, which classified the motifs into eight clusters. All the converted peak information is displayed as scatter plots based on UMAP embedding (Fig. 5 b). The colour code is based on the cluster (Fig. 5 c, left panel) or the ratio of the read depth of the ΔBCL7B group to the control group (Fig. 5 c, right panel). According to the bar graph (Fig. 5c, upper right panel) indicating the ratio of peaks derived from Peaks_2.0 (orange bars) and Pekas_0.5 (blue bars), the clusters 3 and 4, consisted only of high peaks derived from ΔBCL7B. These peaks containing high frequencies of short motifs #13 and #49 (Fig. 5d). On the other hand, Clusters 5 and 7 preferentially consisted of peaks derived from the control group, and their centroid sequences indicated the high frequency of short motifs #79 and #60 (Fig. 5d). Finally, using the RNA-seq data, the genes downregulated in ΔBCL7B group that located within 10 kb of the peaks belonging to clusters 3 and 4 were shown as tables. Interestingly, the immune response-related genes IRF1, CIITA, TAP1, etc., were detected (Fig. 5e). These results indicated that the BCL7B deficient cells undergo the epigenetic change of H3K27me3 marks by recognition sequence changes, and the change impacted immune related genes, especially MHCI and MHCII genes expressions, under control.

## Discussion

Because the concept of cancer stem cells comes from transplantation of a small number of malignant cells in immune-deficient mice [1]. It is still difficult to obtain a lot of cancer stem cells to study. To understand the mechanisms by which BCL7B is involved in cancer pathology, we generated BCL7B-deficient stomach cancer cell lines, and comprehensive gene expression profile of the control cells was compared to the profile of deficient cell lines by RNA-seq. The results showed the downregulation of antigen presentation-related genes in the deficient cell lines; these downregulated genes included IRF1, NLRC5, CIITA, MHC class I, MHC class II and so on (Figs. 2 and 3). Also, these cells were upregulated expression of stemness markers, KRT17, CD99, and so on (Fig. 4). The downregulation of Let-7 miRNAs, malignancy indicator [25, 26] was also shown (Extended Data Table 2). Next, we evaluated the mechanism by which the expression of these genes is changed. Considering the SWI/SNF functions, we performed ChIP-seq analysis to evaluate epigenetic changes. These results indicated that BCL7B-deficient cells underwent changes in H3K27me3 marks at recognition sequences, as indicated by changes in the sequence TTTTT/AAAAA (#79) or TAGAAATA (#60) to AAAAAAAAAANAAAA (#13) or CCCCACCCC (#49), exerted an impact on immune-related genes, especially MHCI and MHCII gene expression in the control group (Fig. 5).

The immune evasion is an intrinsic property of quiescent stem cells in vivo*,* and it results from systematic downregulation of the antigen presentation machinery, including MHC class I proteins [24]. According to these results, an intimate relationship between pluripotency and antigen presentation is suggested. Moreover, this concept is compatible with the relationship between cancer and cancer stem cells. Recently, the stemness of cancer cells was demonstrated by using RNA-seq data obtained from 8290 primary cancer samples representing 21 solid cancer types from The Cancer Genome Atlas (TCGA) [42]. According to the results of this prior study, the properties of stemness were associated with a suppressed immune response, higher intratumoral heterogeneity, and markedly worse outcome [42]. These simple concepts are summarized in Extended Data Fig. 9a.

Additionally, MHCII is a cell surface glycoprotein complex that plays an important role in the adaptive immune response by presenting peptide antigens to CD4^+^ T cells. Recently, the importance of MHCII cell surface expression levels in cancer therapy has been recognized. The reduced expression of the cell surface antigen presentation protein MHCII in hepatocellular carcinoma has been shown to promote progression of cancer [43]. Additionally, the MHCII expression level determines the effectiveness of anti-PD-1/PD-L1 immune checkpoint therapy [44, 45]. These results suggested that it is more difficult to treat cancer cells with low MHCII expression.

In 2018, an integrative network analysis of biological pathways was performed with Williams syndrome patients [46]. According to the results of experiments on peripheral blood, antigen processing, antigen presentation, and B-cell activation were upregulated in the patient samples compared to the control samples. B-cell receptor (BCR) signalling-related genes were included in the modules, with BCL11A found to be the most highly expressed hub gene. BCL11A is a key molecule that promotes B-cell differentiation. Additionally, BCL11B is a key molecule that promotes αβ T-cell differentiation [47]. Thus, we speculated that BCL7B and BCL11A/BCL11B are commonly involved in immune system regulation, although their functions are slightly different. Recently, BCL11A and BCL11B have been reported to be accessory components of the SWI/SNF complex [12]. In our RNA-seq data from Kato III cells, the expression of these molecules was not detected (Extended Data Fig. 8), presumably because the Kato III cell line is derived from stomach cancer cells, which are unrelated to B-cell/T-cell differentiation. By using Kato III cells, which show no functional interference among BCL7B and BCL711A/BCL711B, the function of BCL7B was more easily analysed.

In this study, we revealed that BCL7B, a SWI/SNF chromatin-remodelling complex subunit, is a critical regulator of antigen presentation and stemness in KatoIII cells. BCL7B was expected to show potential as a biomarker for prognosis and immunotherapy analysis due to a correlation found between BCL7B expression and immune cell infiltration in sarcoma [48], and interestingly, the correlation was found to be reversed in certain cancer types [49]. For example, it was reported that the low expression of BCL7B was associated with a poor prognosis in kidney renal clear cell carcinoma (KIRC), kidney renal papillary cell carcinoma (KIRP), skin cutaneous melanoma (SKCM), thyroid carcinoma (THCA), and sarcoma (SARC) [49]. On the other hand, it was reported the high expression of BCL7B was associated with an inferior prognosis in glioblastoma multiforme (GBM), glioma (GBMLGG), kidney chromophobe (KICH), brain lower grade glioma (LGG), oral squamous cell carcinoma (OSCC), rectum adenocarcinoma (READ), and uveal melanoma (UVM) [49]. Although the relationship between BCL7B expression status and poor prognosis was opposite in several cancers, it was also reported that gene set enrichment analysis (GSEA) suggested that BCL7B was notably associated with immune-related pathways in both property-type cancers [49]. Taken together, it is strongly suggested that the BCL7B is one of the master molecules to determine the characteristic immune properties. We would like to propose that other SWI/SNF component molecules, even though we do not know at the moment, contribute to this mechanism, and the combination of SWI/SNF molecules may be a critical to express the opposite functions. Together, these results suggest that BCL7B is a key molecule in immunotherapy and in changes to cell properties. We hope that this result will be helpful in overcoming cancer progression, particularly cancer recurrence due to immune evasion of tumour cells and treatment-resistant cancer stem cells.

## Materials and methods

### Cell cultures

KATO III cells were established in vitro from a pleural effusion of a 55-year-old, Asian male stomach cancer patient, and we obtained KATOIII cells through ATCC. Kato III and ΔBCL7B Kato III cells (See the sentence in establishment of BCL7B-deficient cell lines) were maintained in high-glucose DMEM (043–30085, Wako, Japan) containing 10% FBS (26,140–079, Gibco, Gaithersburg, MD), 1% penicillin‒streptomycin (P4333, Sigma‒Aldrich, St. Louis, MO). U937 and U937ΔBCL7B cells were maintained in RPMI-1640 (189–02025, Wako, Japan) containing 10% FBS (26,140–079, Gibco, Gaithersburg, MD) and 1% penicillin‒streptomycin (P4333, Sigma‒Aldrich, St. Louis, MO).

### Generation of BCL7B genome editing CRISPR/Cas9 plasmid vectors and establishment of BCL7B-deficient cell lines

A schematic overview showing the experimental design is presented in Fig. 1a. We used CRISPR/Cas9 in one commercial vector system (Cas9 SmartNuclease™ All-in-one Vector, SBI System Biosciences, CA, U.S.A.). BCL7B in *Homo sapiens* is encoded into three isoforms. These isoforms share a common transcription initiation sequence region that extends to the first methionine undergoing translation. Two guide RNAs (gRNAs) for each CRISPR/Cas9 genome-editing system were designed for the two regions (Extended Data Fig. 1a). The two constructed CRISPR/Cas9 vectors, including each gRNA sequence, were transfected with concatenated PCR products of the regions before and after the gRNA sequences for homology-directed repair (HDR) (Extended Data Fig. 1, b). One day before transfection, Kato III cells were seeded in 35-mm dishes (2 × 10^5^ cells/dish). The cells were transfected with the two targeting BCL7B gene CRISPR/Cas9-edited plasmids (0.5 μg each) and PCR products for HDR (1 μg) using Lipofectamine 2000 (11,668,027, Thermo Fisher Scientific, MA, U.S.A.) according to the manufacturer’s instruction manual. For U937 cells, Amaxa® nucleofector® Kit C (Lonza, Basel, Switzerland) was used for transfection. Twenty-four hours after transfection, these cells were seeded at a limited concentration (0.5–5 cells/well in each plate) in 96-well plates. A few weeks later, some of the cells in one well were harvested, and PCR was performed to confirm the sequence that was deleted. The populations including deficient cells were seeded again in limited concentrations (0.5 cells/well each plate) in 96-well plates, and the cell number was counted to confirm that a single cell was present in each well under a microscope. A few weeks later, some of the cells in one well were harvested, PCR was performed again to identify the deleted sequence. After the limited concentrations of cells were seeded and assessed of a few more times, the candidate BCL7B-deficient cell lines were established by assessing the BCL7B-deficient sequence (Extended Data Fig. 1c). The candidate cell lines were expanded for a few months, and some of the cells were harvested for quantitative real-time PCR to measure the expression level of the BCL7B gene. Then, we established three BCL7B-deficient cell lines (BCL7BΔ1-3 cells), which showed a significant decrease in BCL7B expression (Fig. 1c).

### Quantitative real-time PCR

RNA was isolated using an RNeasy mini kit (74,104, Qiagen, Germany), and cDNA was synthesized using a SuperScript™ IV First-Strand cDNA Synthesis System (18,091,050, Thermo Fisher Scientific, MA, U.S.A.). The cDNA was used for quantitative real-time polymerase chain reaction (PCR) analyses. The analyses were performed using target gene primers corresponding to the following *Homo Sapiens* sequences: GAPDH (forward, 5’ TGCACCACCAACTGCTTAGC; reverse, 5’ GGCATGGACTGTGGTCATGAG); BCL7A (forward, 5’ CACCCAGGAGCTGAAGATGC; reverse, 5’ TTTCTCTGAGCTGTTCATCG); BCL7B (forward, 5’ GTGGGTGACACGTCCCTGAGG; reverse, 5’ GGCTGCTGAACTGTTCGATT); BCL7C (forward, 5’ CTGGGCCAAGAGAGAGATCC; reverse, 5’ CTTCCAGCAGTTCTGGAACA); NLRC5 (forward, 5’ CCTAGAGGAGCTGGACTTGAG; reverse, 5’ TGCCAACTGCACTCCCCCGG); CIITA (forward, 5’ AGAAACTGGAGTTTGCGCTG; reverse, 5’ TTGAGGGTTTCCAAGGACTT; CASP1 (forward, 5’ TCTCACTGCTTCGGACATG; reverse, 5’ CAGGAACGTGCTGTCAGAGG); IRF1 (forward, 5’ CAAGGCCAAGAGGAAGTCAT; reverse, 5’ CTGTGGTCATCAGGCAGAGT); PSMB9 (forward, 5’ AGCTGGAGCTCCATGGGATA; reverse, 5’ CCAGCCAGCTACCATGAGAT); and TAP1 (forward, 5’ GTCTTAGTGCTACAGGGGCTG; reverse, 5’ CTGCCTGTGCAGGTAGCGGT). A 25-µl aliquot of real-time PCR mixture including 1 × SYBR Green PCR Master Mix (4,367,659, Applied Biosystems, UK), gene-specific primers (0.4 µM), and cDNA template (300 ng) was run on a 7500 Real-Time PCR system (Applied Biosystems, UK). The PCR conditions were as follows: 95 °C for 10 min, then 40 cycles at 95 °C for 5 s, 58 °C for 10 s, and 72 °C for 34 s.

### RNA sequencing

Total RNA was isolated using an RNeasy mini kit (74,104, Qiagen, Germany). After polyA enrichment (Dynabeads™ mRNA DIRECT™ Micro Purification Kit, 61,021, Thermo Fisher Scientific, MA, U.S.A.), a library for RNA-seq was prepared according to Ion Torrent protocols (Ion Total RNA-seq Kit for the AB Library Builder™ System; P/N 4482416, Ion Xpress™ RNA-Seq Barcode 01–16 Kit; P/N 4475485, Ion Total RNA-seq Protocol Card for the AB Library Builder™ System; P/N 4482563, Agilent® High Sensitivity DNA Kit; 5067–4626; Agilent®RNA6000 pico Kit; 5067–1513, Agilent®RNA6000 pico ladder; 5067–1535, Thermo Fisher scientific, MA, U.S.A.) on an Ion Torrent Ion Chef/Ion Proton system (Thermo Fisher Scientific, MA, U.S.A.). The fragments per kilobase of exon per million reads mapped (FPKM) values were calculated by the RNA-seq analysis plugin in TorrentSuite (https://github.com/iontorrent/TS).

### Gene Set Enrichment Analysis (GSEA) and Gene Ontology (GO)

GSEA and GO analysis were performed as previously described  [50]. NGS data were examined and visualized using R/Bioconductor [51]. NGS mapping and postprocessing for RNA-Seq data analysis were performed with the Bioconductor package QuasR [52], which uses Rbowtie for ungapped alignment and SpliceMap for spliced alignment. Gene level numbers were determined by qCount, and simple RPKM normalization was performed. Investigation of the ontology of differentially expressed genes was achieved as previously reported [53]. The statistical significance of the gene expression of individual genes was determined as a value of *P* < 0.05 after Welch’s ANOVA, which was used to compare the average normalized signal using the Bioconductor package Genefilter. To investigate the ontology of the extracted genes, GO terms enriched with expressed genes were assessed by hypergeometric test [54] using GOstats. The result from the hypergeometric test for a given GO term in a pairwise comparison group is two pairs of *P* values: increase.overrepresented vs. increase.underrepresented and decrease.overrepresented vs. decrease.underrepresented. Only a term with overrepresented *P* values lower than the corresponding underrepresented *P* value was selected. GO terms were extracted based on a *P* value threshold of 10^5^ and visualized in a heatmap. For the heatmap, P values showing an increase or decrease in the hypergeometric test for a given GO term-enriched gene in each pairwise comparison group was visualized by red or blue, respectively. Hierarchical clustering was performed using Ward’s method to calculate linkage distances based on the correlation coefficient between samples or GO terms.

### ChIP-seq

ChIP-seq libraries for use in the Ion Proton system were prepared according to Ion Torrent protocols, user bulletin publication Number 4473623 revision B (MAGnify™ Chromatin Immunoprecipitation System; Thermo Fisher Scientific 492,024, Ion Xpress™ Plus Fragment Library Kit; Thermo Fisher Scientific 4,471,269, Ion Xpress™ Barcode Adapters 1–96 Kit; Thermo Fisher Scientific 4,474,517). Briefly, cells were fixed with 1% paraformaldehyde for 10 min at room temperature. Approximately 2 × 10^6^ cells per sample were sonicated on a Covaris® S2 system to shear chromatin (microtube snap-cap, 520,045, M&S instruments Inc.). The program of the Covaris® S2 instrument was as follows: duty cycle, 5%; intensity, 2; cycles per burst, 200; cycle time, 60 s; cycles, 20; temperature (bath), 4 °C; power mode, frequency sweeping; and degassing mode, continuous. For each immunoprecipitation (IP) reaction, 25 μg of the sheared chromatin and 5 μg of a mouse monoclonal anti-H3K27me3 antibody (ChIP Grade ab6002, Abcam) were used. After overnight reaction at 4 °C, the Chromatin-Antibody-Dynabeads® complexes were isolated, and the crosslinking was reversed to purify the DNA. The ends of the purified ChIP-DNA were repaired and ligated to barcode adapters. The samples were nick repaired to complete the linkage between adapters, and the DNA was amplified by PCR. Finally, the libraries were purified to select fragments approximately 160–340 bp in length. The DNA libraries were diluted to 20 pM by LowTE and sequenced using an Ion Chef/Ion Proton system (Thermo Fisher Scientific, Waltham, MA, U.S.A.) according to the standard protocol. Then, the sequences of the DNA fragments were mapped to the human genome (hg19 and GRCh38.p12) using Ion Torrent Suite (ver. 5.14.0, https://github.com/iontorrent/TS).

### Data analysis of ChIP-seq data and motif identification

Sequences of the DNA fragments obtained by chromatin immunoprecipitation and next-generation sequencing of the parent cell line and the ΔBCL7B cell lines were subjected to the following analysis to determine the short motif sequences that appeared specifically in each sample. First, we calculated a moving average of the read depth across the entire genome and looked for minima and maxima, as well as points where the slope changed more than twofold. Based on the positional relationship of these points, we exhaustively extracted the combinations of the start, maximum, and end positions of the peaks. For these combinations, the average read depths at the start and end positions were used as baselines, and regions where the maximum read depth was greater than 2.5 and the integrated read depth from start to end was greater than 5.0 were considered candidate peak regions. For each peak candidate, the average depth values were calculated for the parent cell line ([Dp]_ctrl) and each ΔBCL7B cell line ([Dp]_∆BCL7). Then, we compared the average depths and identified 372,870 regions in which [Dp]_ctrl is lower than one-half of the [Dp]_∆BCL7, as Peaks_2.0, and 367,631 regions, in which the [Dp]_ctrl was larger than twice that of the [Dp]_∆BCL7, as Peaks_0.5. The depths of reads and the positions of peaks were visualized by IGV [55]. Next, we listed the DNA sequences of the peak regions and used them for motif identification using STREME [56]. We found 92 and 113 motifs from Peaks_2.0 and Peaks_0.5, respectively. Since some of these motifs were homologous, we treated them as identical motifs and created a consolidated list of motifs. Using this list, we examined the frequency of each motif in all the detected peaks and converted the peaks to frequency vectors. These vectors were used to classify the data into eight clusters by k-means. Based on the proportion of vectors derived from Peaks_2.0 and Peaks_0.5 in each cluster, clusters that preferentially included vectors derived from Peaks_2.0 and vectors derived from Peaks_0.5 were selected. Finally, based on the centroids of these clusters, the short motif sequences with a high frequency as determined by ChIP were identified for each sample of the parental and ΔBCL7B cell lines. For machine learning and visualization of the results, we used scikit-learn and a UMAP library [57, 58]. To perform the computational calculations, we ran our original C +  + codes and Python scripts (https://github.com/YujiSue/Research/tree/main/CustomChIP) at our workstation CELSIUS R940 (Fujitsu, Tokyo) and Google Colab [59].

### Flow cytometry analysis

Cells were harvested and washed with PBS. They were filtered through a 40-μm cell strainer and immediately sorted with a MoFlo Astrios EQ (Beckman Coulter Inc., California, U.S.A.).

### Immunohistochemistry

Cultured cells were washed once with PBS and fixed in 4% paraformaldehyde (Electron Microscopy Sciences) for 10 min at RT. Samples were washed twice with PBS, and 0.1% Triton-X solution was added to promote membrane permeability by the antibody against cytokeratin 17. The samples were washed three times and then treated with CAS-Block solution (008120, Thermo Fisher Scientific, MA, U.S.A.) for 30 min at RT. Primary antibodies were diluted with CAS-Block solution and then applied to the samples, and the treated samples were incubated at 4 °C overnight. We used the following antibodies at a 1:100 dilution: anti-cytokeratin 17 antibody (ab53707, Abcam) and anti-CD99 antibody [EPR3096] (ab108297, Abcam), a 1:50 dilution: EZH2 antibody (21,800–1-AP, proteintech). After rinsing four times, for 15 min each time, in PBS, the samples were incubated at room temperature for 3 h with fluorescent secondary antibodies (Alexa-488, Invitrogen, CA, U.S.A. or CoraLite488, proteintech, IL, U.S.A.). The samples were observed under a fluorescence microscope after four final rinses, each for 15 min each time, in PBS.

### RNA interference in C. elegans

Several mutant animals, listed in Extended Data Fig. 4a, were obtained from the C. elegans Genetic Center. RNA interference analyses (RNAi) were performed by feeding animals dsRNA-producing bacteria as described previously [60]. Briefly, the control and bcl-7 RNAi clones were obtained from the Ahringer RNAi library, and P0 animals in the early L1 stage were transferred to plates containing RNAi bacteria grown on 100 µg/mL ampicillin and 1 mmol/L isopropyl-beta-D-thiogalactopyranoside (IPTG). The F1 animals were cultured at 20 °C until they reached adulthood. The early-stage L4 adult animals, which were treated with control siRNA and bcl-7 siRNA, were observed under a fluorescence microscope. Images were obtained with a DP80BW CCD camera.

### Statistical analysis

Statistical analyses were performed by Student’s t test.

### Supplementary Information

**Additional file 1.**

**Additional file 2.**

## Data Availability

The data that support the findings of this study are openly available in NCBI SRA at https://www.ncbi.nlm.nih.gov/sra, RNA-seq accession number; SRR24463223, SRR24463222, SRR24463213, SRR24463212, SRR24463211, SRR24463210, SRR24463209, SRR24463208, ChIP-seq accession number; SRR24463207, SRR24463206, SRR24463221, SRR24463220, SRR24463219, SRR24463218, SRR24463217, SRR24463216, SRR24463215, SRR24463214.
