# Supplementary material for: BCL7B, a SWI/SNF complex subunit, orchestrates cancer immunity and stemness
Source: BMC Cancer. 2023 Aug 30;23:811. doi: 10.1186/s12885-023-11321-3 (PMC10466690; doi:10.1186/s12885-023-11321-3)
Supplement: Supplementary file 1 — Additional file 1. [file 12885_2023_11321_MOESM1_ESM.pdf]

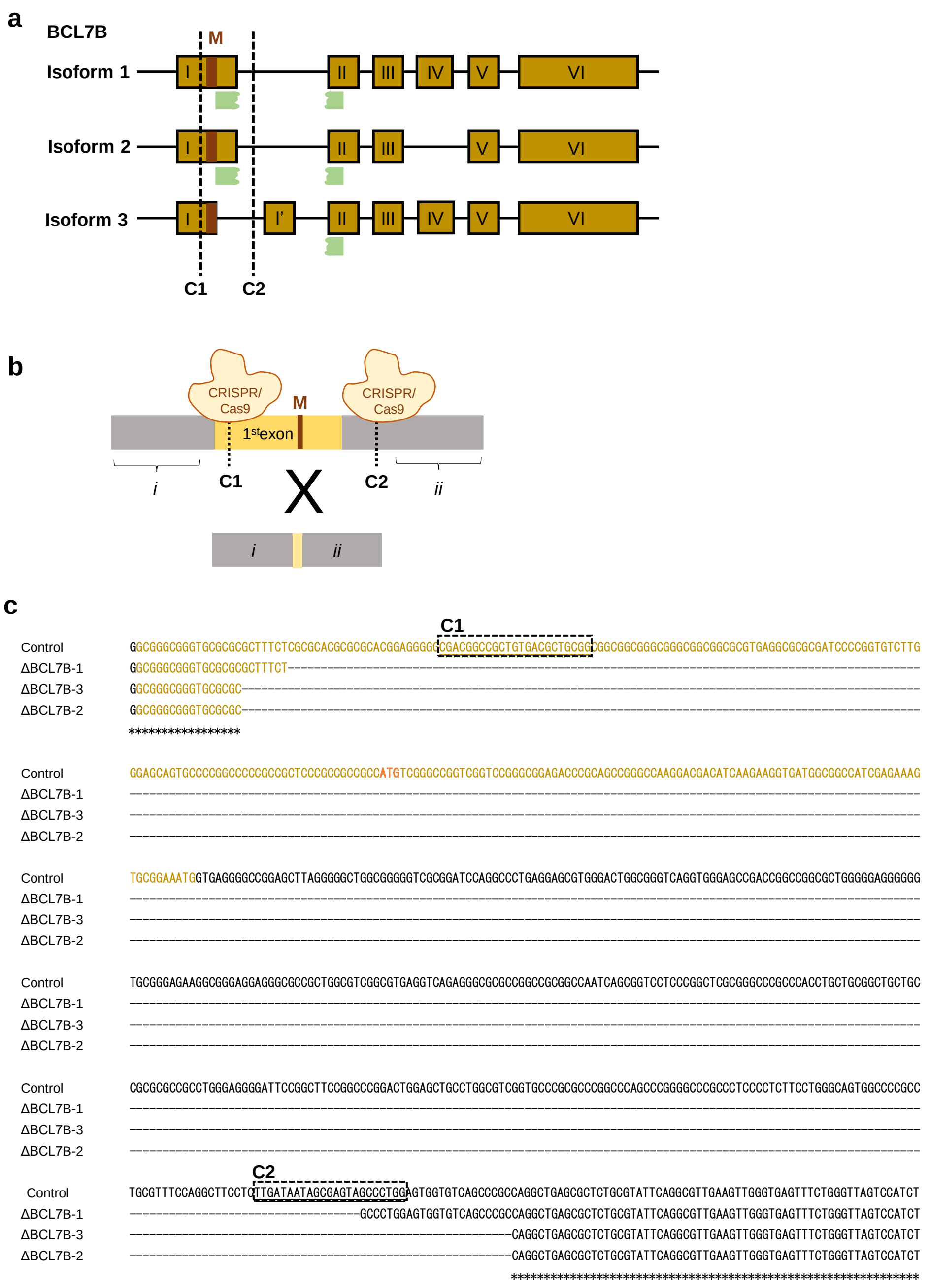

Extended Data Figure 1

(a) Schematic representation of the BCL7B gene. The yellow columns indicate the exon regions, and black lines indicate intron regions. The brown lines indicate the first methionine (M). The green columns under the first (I) and the second (II) exons indicate the conserved N -terminal region in BCL family genes. The dotted lines indicate the two target sites of single-guide RNAs used with the CRISPR/Cas9 editing system (C1 and C2). (b) Schematic representation showing BCL7B genome editing by the CRISPR/Cas9 system. The yellow columns indicate the first exon, and the grey columns indicate the 5' UTR and the intron regions. The brown line indicates the first methionine (M). The dotted lines indicate the two target sites of single guide RNAs used in the CRISPR/Cas9 editing system (C1 and C2). The two CRISPR/Cas9 vectors, including each gRNA sequence, were transfected with a concatenated regions obtained by PCR before and after gRNA (i and ii) for homology-directed repair (HDR). (c) The deleted portions of sequences in each cell line are shown. The dotted columns (C1 and C2) indicate the two reference sequences used for the single-guide RNAs. The yellow sequences indicate exon sequences, and the first methionine codon is shown in orange. The black sequences indicate introns.

Chr. No.	Ensemble gene_id	Gene short name	Δ1 ratio av	Δ2 ratio av	Δ3 ratio av	Average
6p21.32	ENSG00000196735.7	HLA-DQA1	0.038787643	0.055119322	0.09831524	0.064074068
6p21.32	ENSG00000237541.3	HLA-DQA2	0.066672642	0.053129569	0.087528601	0.06911027
11q22.3	ENSG00000137752.18	CASP1	0.023335393	0.079072025	0.118327848	0.073578422
Gene Cluster	ENSG00000252974.1	AC121334.1	0	0	0.224357108	0.074785703
5q31.2	ENSG00000206989.1	SNORD63	0.032814485	0.392930822	2.82574E-09	0.141915103
1p22.2	ENSG00000162654.8	GBP4	0.098847646	0.055599962	0.271787081	0.14207823
7p15.2	ENSG00000207584.1	MIR196B	0.016164287	0.50321847	0	0.173127586
8q24.3	ENSG00000216133.1	MIR939	0	0.60650881	0	0.202169603
6p21.32	ENSG00000204252.8	HLA-DOA	0.103939933	0.109823225	0.392804326	0.202189161
10q23.31	ENSG00000119917.9	IFIT3	0.152096931	0.299024956	0.174643582	0.208588489
3q25.32	ENSG00000118849.5	RARRES1	0.35992374	0.132262672	0.173406567	0.221864326
6p21.32	ENSG00000232629.4	HLA-DQB2	0.153266071	0.211547383	0.305352567	0.223388674
22q12.3	ENSG00000128284.15	APOL3	0.157379992	0.245391612	0.306406394	0.236392666
1p22.2	ENSG00000117228.9	GBP1	0.150778343	0.234998776	0.33264906	0.239475393
7q34	ENSG00000204983.8	PRSS1	0.430386953	0.280801454	0.014975571	0.242054659
?	ENSG00000265039.1	AC107016.2	0.15694436	0.389331517	0.185547391	0.24394109
8p11.21	ENSG00000131203.8	IDO1	0.161848524	0.008930665	0.587299252	0.252692814
16p12.3	ENSG00000005187.7	ACSM3	0.399865898	0.12520092	0.241076069	0.255380962
6p21.32	ENSG00000198502.5	HLA-DRB5	0.208207556	0.20203604	0.378089852	0.262777816
6p21.32	ENSG00000223865.6	HLA-DPB1	0.182416609	0.290558468	0.336604771	0.269859949
22q12.3	ENSG00000221963.5	APOL6	0.237181967	0.421307576	0.181636166	0.280041903
9q22.32	ENSG00000199072.1	MIRLET7F1	0.316251078	0	0.5279869	0.281412659
6p21.32	ENSG00000231389.3	HLA-DPA1	0.188889638	0.198343709	0.459001311	0.282078219
5q35.3	ENSG00000221394.1	MIR1229	0.636622135	0.160133989	0.055359017	0.28403838
9q22.32	ENSG00000199133.1	MIRLET7D	0.267866335	0.484002986	0.105284774	0.285718032
14q32.2	ENSG00000140105.13	WARS	0.332397309	0.344053206	0.185520567	0.287323694
6p21.32	ENSG00000179344.12	HLA-DQB1	0.213062446	0.309477549	0.35286438	0.291801458
17p13.1	ENSG00000132530.12	XAF1	0.267420844	0.399652534	0.224127971	0.297067116
16p13.13	ENSG00000179583.13	CIITA	0.211542183	0.319080772	0.3757944	0.302139118
22q12.3	ENSG00000100342.16	APOL1	0.154774407	0.367001821	0.419846838	0.313874355
16q24.3	ENSG00000265672.1	MIR4722	0.768049857	0.202708519	0	0.323586125
6p21.32	ENSG00000242574.4	HLA-DMB	0.35950538	0.259809961	0.351565011	0.323626784
22q12.3	ENSG00000128335.9	APOL2	0.182004143	0.554844975	0.244871342	0.327240153
6p21.32	ENSG00000229391.3	HLA-DRB6	0.133916821	0.274191087	0.588464363	0.332190757
MT	ENSG00000210164.1	MT-TG	0.210464595	0.546034829	0.243547138	0.333348854
9q21.32-q21.33	ENSG00000197506.6	SLC28A3	0.444039151	0.418300142	0.141834996	0.334724763
6p21.32	ENSG00000204287.9	HLA-DRA	0.239332571	0.289114284	0.481405764	0.33661754
17q11.2	ENSG00000238649.1	SNORD42A	0.145087886	0.160375556	0.713096139	0.33951986
22q13.31	ENSG00000198986.1	MIRLET7A3	0.295155589	0.249848099	0.485827908	0.343610532
13q14.11	ENSG00000133106.10	EPSTI1	0.304452705	0.35371436	0.406730875	0.35496598
16q23.3	ENSG00000103154.5	NECAB2	0.316461877	0.707208886	0.042457241	0.355376001
2q24.2	ENSG00000115267.5	IFIH1	0.296051201	0.471443322	0.320131097	0.362541874
MT	ENSG00000210156.1	MT-TK	0.21015503	0.719754801	0.189498544	0.373136125
5q33.1	ENSG00000019582.10	CD74	0.373938208	0.294002027	0.481235183	0.383058473
6p21.32	ENSG00000204257.10	HLA-DMA	0.350021097	0.406427398	0.393914997	0.383454497
7q22.3	ENSG00000164597.9	COG5	0.318438016	0.600630854	0.236223182	0.385097351
5q31.1	ENSG00000125347.9	IRF1	0.299924463	0.537081323	0.354669354	0.397225046
11q12.3	ENSG00000133321.6	RARRES3	0.381724088	0.4853108	0.329186915	0.398740601
17q21.31	ENSG00000068079.3	IFI35	0.359175673	0.601059787	0.236895815	0.399043758
17q21.31	ENSG00000235378.2	MRPS10P1	0.617729486	0.410537492	0.177998995	0.402088658
21q22.3	ENSG00000157601.9	MX1	0.383374597	0.517709158	0.319446151	0.406843302
6p21.32	ENSG00000196126.6	HLA-DRB1	0.283315766	0.328369944	0.614170364	0.408618691
7q32.1	ENSG00000158467.12	AHCYL2	0.658575794	0.458577354	0.13063648	0.415929876
9q34.3	ENSG00000127191.13	TRAF2	0.380941016	0.238863046	0.633720721	0.417841595
?	ENSG00000237875.1	RP5-1174J21.1	0.631194952	0.299977367	0.337119266	0.422763862
5q35.3	ENSG00000264549.1	SNORD95	0.367379883	0.372374013	0.536467572	0.425407156
5q31.1	ENSG00000197536.6	C5orf56	0.326842521	0.673398535	0.278792309	0.426344455
6p21.1	ENSG00000265700.1	MIR4647	0.444471112	0.408269402	0.440599945	0.431113487
7q22.1	ENSG00000160862.8	AZGP1	0.396393457	0.691095108	0.243486148	0.443658238
19q13.33	ENSG00000105550.4	FGF21	0.843061037	0.450835708	0.039608953	0.444501899
3q21.1	ENSG00000163840.5	DTX3L	0.430467178	0.537555973	0.372088749	0.446703967
16p13.2-16p13.13	ENSG00000166669.9	ATF7IP2	0.342823256	0.471622744	0.54387409	0.452773363
1p34.1	ENSG00000264294.1	SNORD55	0.300413979	0.488623349	0.578805725	0.455947684
9q22.32	ENSG00000199165.2	MIRLET7A1	0.337023857	0.414920251	0.641434377	0.464459495
6p21.31	ENSG00000010030.9	ETV7	0.432043883	0.474617651	0.530792461	0.479151331
17q25.3	ENSG00000173821.15	RNF213	0.49368973	0.373682121	0.575236945	0.480869599
16p13.3	ENSG00000103355.8	PRSS33	0.260740526	0.70290992	0.483954116	0.482534854
4q35.1-q35.2	ENSG00000145476.11	CYP4V2	0.796355295	0.467523177	0.188567491	0.484148654
3q21.1	ENSG00000173193.9	PARP14	0.483236218	0.495216172	0.480858417	0.486436936
7q34	ENSG00000105939.8	ZC3HAV1	0.662802056	0.444805415	0.355586226	0.487731232
14q12	ENSG00000100911.9	PSME2	0.407054869	0.594921904	0.463809467	0.488595413
7q22.1	ENSG00000169871.8	TRIM56	0.779643646	0.341407691	0.353827776	0.491626371
6p21.32	ENSG00000168394.9	TAP1	0.443142146	0.479575614	0.561956163	0.494891308
6p21.32	ENSG00000240065.3	PSMB9	0.456297782	0.619913439	0.417822392	0.498011204
3q25.33	ENSG00000213186.3	TRIM59	0.722958101	0.294303103	0.490999483	0.502753563
6p21.33	ENSG00000234745.5	HLA-B	0.441365124	0.580693188	0.493663225	0.505240512
1p36.32	ENSG00000157873.13	TNFRSF14	0.457502777	0.708717601	0.351348511	0.505856296
7q31.32	ENSG00000128609.10	NDUFA5	0.457871502	0.733485629	0.351568842	0.514308658
16q13	ENSG00000140853.11	NLRC5	0.388651957	0.742178657	0.419869042	0.516899885
4q35.1	ENSG00000109794.9	FAM149A	0.744907699	0.426872154	0.388918526	0.520232793
22q12.2	ENSG00000239127.1	SNORD125	0.368506312	0.844477996	0.358132533	0.523705614
7q11.23	ENSG00000106635.3	BCL7B	0.752849563	0.404549721	0.423954564	0.52711795
6p22.1	ENSG00000204592.5	HLA-E	0.453795105	0.674738249	0.459107259	0.529213537
?	ENSG00000256929.1	AC067852.1	0.371899134	0.857030796	0.362198605	0.530376179
8q22.3	ENSG00000048392.7	RRM2B	0.493075675	0.407824776	0.70717733	0.536025927
4q34.3	ENSG00000038002.4	AGA	0.766673367	0.47972774	0.397427926	0.547943011
11p15.4	ENSG00000132109.8	TRIM21	0.427222522	0.819799949	0.453373612	0.566798694
Xq23	ENSG00000101935.5	AMMECR1	0.45075928	0.830295106	0.429716248	0.570256878
22q12.1	ENSG00000100219.12	XBP1	0.430167713	0.822471449	0.458829277	0.57048948
7q31.1	ENSG00000177683.9	THAP5	0.816270249	0.437023702	0.459257184	0.570850378
?	ENSG00000226624.1	CTA-351J1.1	0.448635589	0.466187827	0.808445552	0.574422989
22q13.1	ENSG00000128298.12	BAIAP2L2	0.480965214	0.867644296	0.432646766	0.593752092

Extended Data Table 1

The list of genes indicates the downregulated mRNAs in all three BCL7B-deficient cell lines compared to the control Kato III cell line, as shown in ascending order of the average ratio.

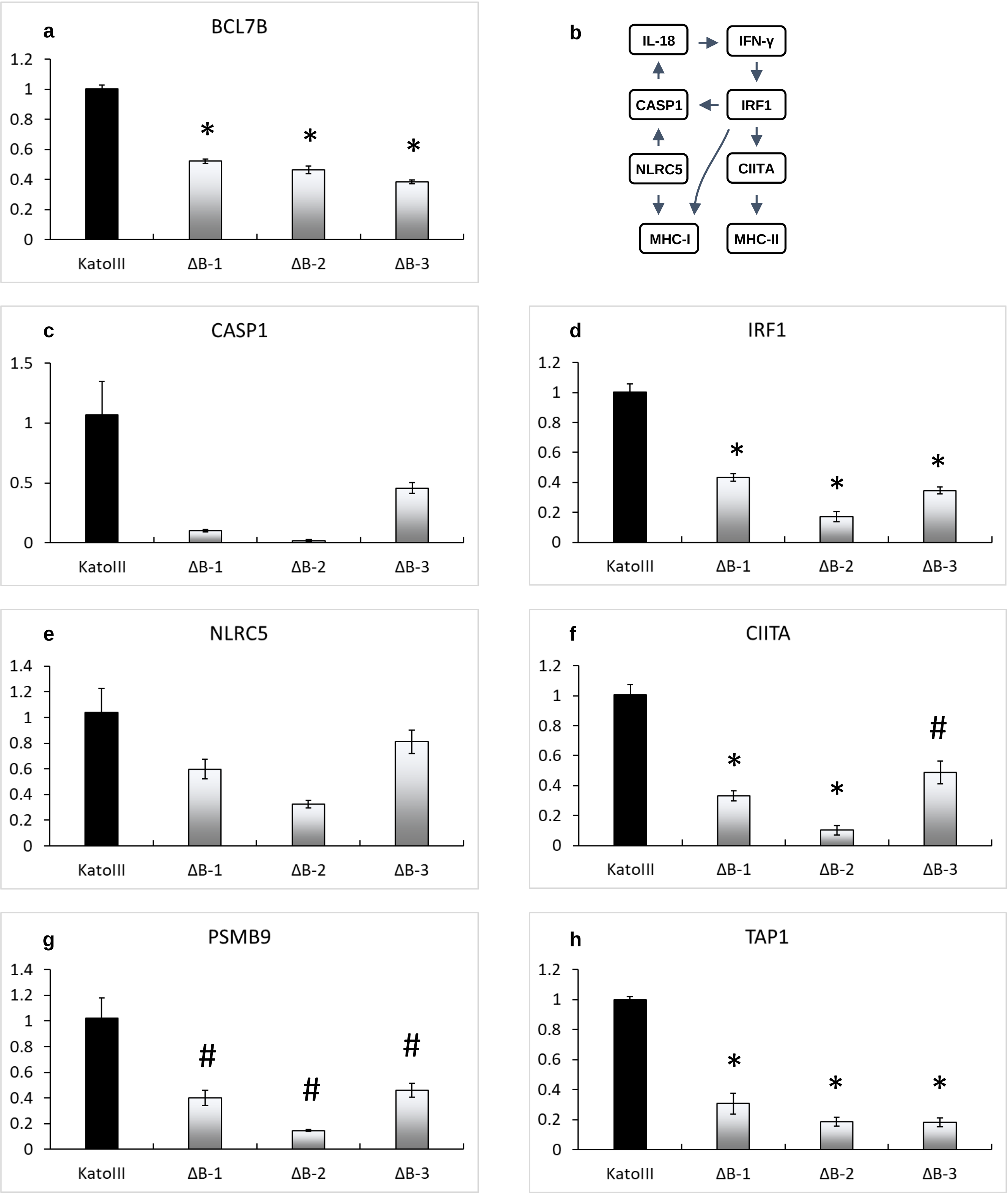

Extended Data Figure 2

The comparison of gene expression levels between KatoIII and BCL7B deficient cells by qPCR. (a) The expression levels of BCL7B were determined by qPCR and were reported relative to that in control cells. (b) Simplified diagram showing the relationships of antigen presentation molecules. (c-h) The expression levels of the antigen-presentation related genes were determined by qPCR and were reported relative to that in control cells. (a, c-h) SE (n = 3). The symbols show the statistical significance (*p < 0.005). (*p < 0.005, #p < 0.05, §p < 0.01).

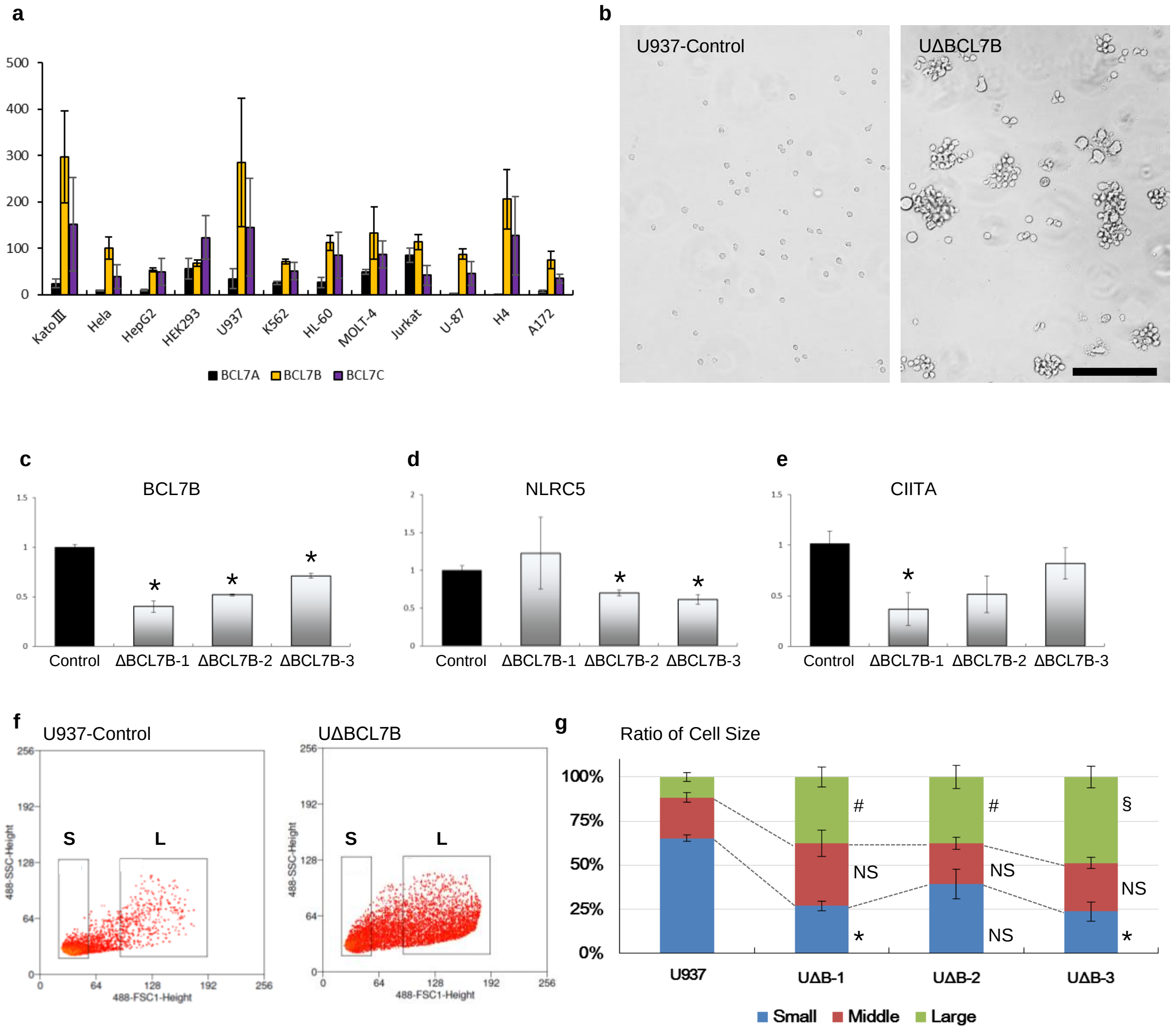

Extended Data Figure 3

(a) The expression levels of the BCL7 family genes (BCL7A, BCL7B and BCL7C) in the several cell lines shown under the bars, as determined by qPCR. The BCL7A abundance in H4 cells was set to be equal to 1. SE (n = 2). The BCL7B expression levels were high in Kato III and U937 cells. Error bars represent the SEMs. (b-e) Establishment of BCL7B-deficient U937 cell lines and the expression levels of the BCL7B, NLRC5 and CIITA genes. (b) Phase contrast images of cultured cells. Scale bar, 200 μm. (c) The expression levels of the BCL7B gene were determined by qPCR and are reported relative to that in control cells. SE (n = 3). The symbols show the statistical significance (*p < 0.005). (d) The expression levels of the NLRC5 gene were determined by qPCR and are reported relative to that in control cells. SE (n = 3). The symbols show the statistical significance (*p < 0.005). (e) The expression levels of the CIITA gene were determined by qPCR and are reported relative to those in the control cells. SE (n = 3). The symbols show the statistical significance (*p < 0.005). (f) Comparison of cell sizes by flow cytometry. Abbreviations, S; small, L; large. (g) The relative population of small, middle and large cells. Each coloured column indicates the following information: blue bars, small cells; red bars, medium-sized cells; right green bars, large cells. SE (n = 3). The symbols show the statistical significance (*p < 0.005, #p < 0.05, §p < 0.01). Abbreviations, NS; no significance.

a

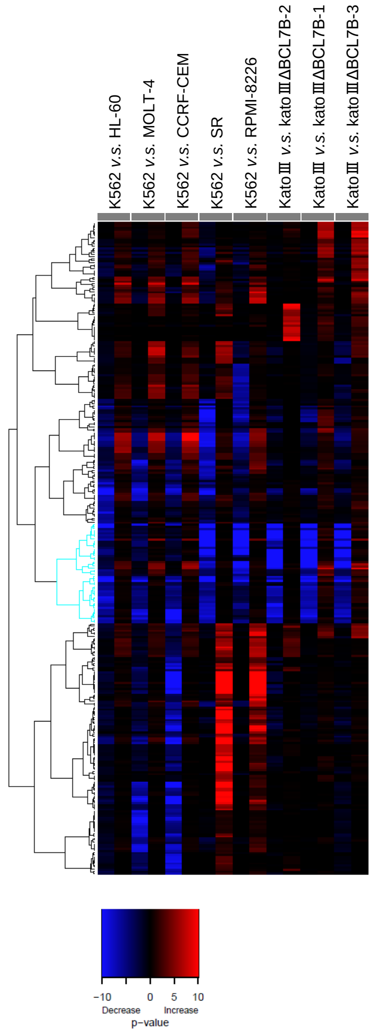

b

ID	GO Term
GO:0006955	immune response
GO:0006952	defense response
GO:0048584	positive regulation of response to stimulus
GO:0071345	cellular response to cytokine stimulus
GO:0019221	cytokine-mediated signaling pathway
GO:0034097	response to cytokine stimulus
GO:0045087	innate immune response
GO:0006950	response to stress
GO:0071357	cellular response to type I interferon
GO:0060337	type I interferon-mediated signalling pathway
GO:0034340	response to type I interferon
GO:0009615	response to virus
GO:0071345	cellular response to cytokine stimulus
GO:0034341	response to interferon-gamma
GO:0060333	interferon-gamma-mediated signaling pathway
GO:0019882	antigen processing and presentation
GO:0031295	T cell costimulation
GO:0031294	lymphocyte costimulation
GO:0071310	cellular response to organic substance
GO:0070887	cellular response to chemical stimulus
GO:0010033	response to organic substance
GO:0042221	response to chemical stimulus
GO:0051707	response to other organism
GO:0009607	response to biotic stimulus
GO:0051704	multi-organism process
GO:0050776	regulation of immune response
GO:0002682	regulation of immune system process
GO:0002376	immune system process
GO:0048002	antigen processing and presentation of exogenous peptide antigen
GO:0002474	antigen processing and presentation of peptide antigen via MHC class I
GO:0050870	positive regulation of T cell activation
GO:0050863	regulation of T cell activation
GO:0050867	positive regulation of cell activation
GO:0002696	positive regulation of leukocyte activation
GO:0051251	positive regulation of lymphocyte activation
GO:0050865	regulation of cell activation
GO:0002694	regulation of leukocyte activation
GO:0051249	regulation of lymphocyte activation
GO:0050778	positive regulation of immune response
GO:0002253	activation of immune response
GO:0002684	positive regulation of immune system process
GO:0002768	immune response-regulating cell surface receptor signaling pathway
GO:0002429	immune response-activating cell surface receptor signaling pathway
GO:0050851	antigen receptor-mediated signaling pathway
GO:0050852	T cell receptor signaling pathway
GO:0042110	T cell activation
GO:0002764	immune response-regulating signaling pathway
GO:0002757	immune response-activating signal transduction

Extended Data Figure 4

Comparison of gene expression profiles between chronic myeloblastic leukaemia (K562) cells and acute leukaemia (HL-60, MOLT-4, CCRF-CEM, SR, RPMI-8226) cells and between the Kato III cell line and BCL7B-deficient Kato III cell lines. (a) Gene set enrichment analysis (GSEA). The heatmap colour scale is shown at the bottom. The expression of the majority of the genes in the blue cluster was downregulated. Each sample is listed in two lines: the left line indicates downregulated genes, and the right line indicates upregulated genes. (b) The Gene Ontology (GO) terms in the blue cluster shown in (a) were related mainly to immunity.

a

Chr. No.	Ensemble gene_id	Gene short name	Δ1-3 ratio Average
7p15.2	ENSG00000207584.1	MIR196B	0.173127586
8q24.3	ENSG00000216133.1	MIR939	0.202169603
9q22.32	ENSG00000199072.1	MIRLET7F1	0.281412659
5q35.3	ENSG00000221394.1	MIR1229	0.28403838
9q22.32	ENSG00000199133.1	MIRLET7D	0.285718032
16q24.3	ENSG00000265672.1	MIR4722	0.323586125
22q13.31	ENSG00000198986.1	MIRLET7A3	0.343610532
6p21.1	ENSG00000265700.1	MIR4647	0.431113487
9q22.32	ENSG00000199165.2	MIRLET7A1	0.464459495

b

miRNA predicted targets (Score ≥95)

Gene short name	Gene short name	Gene short name	Gene short name
RIMKLB	ARIH1	SLC18A2	SH3GL3
RICTOR	ITCH	NDC1	DCAF7
CDK19	SORBS1	MARCH6	SDC2
FAM3C	HMGXB4	BTF3L4	CBLN4
SRSF2	CEP76	B3GNT2	ZNF652
EMP2	CAMTA1	F3	KLF4
BTAF1	HMGCR	ARRDC3	FZD6
PUM2	ARHGAP20	CCNE2	SLC23A2
DAAM1	GLS	PTBP3	DLL4
FRS2	GNB4	FBXO43	SOWAHC
SNAPC1	TRPM7	BDNF	DCAF6
SMARCA5	PAK2	GTF2A1	PDS5B
CCNA2	DCLK3	SHOC2	EEA1
TMEM2	ERF	PPP1R15B	ASAH1
NUDT4	TAOK1	SGTB	DOPEY1
DCUN1D4	WASF3	RDX	ANKRD44
TRIM63	SYF2	CCNY	ADAMTSL3
PAN3	HNRNPD	WDHD1	ALDH1L2
CLOCK	FYTTD1	DLL1	GTF3C3
TSHZ3	NASP	MBLAC2	SPAST
SMEK2	ZCCHC11	ITM2B	SP4
REV3L	CACNA1C	LPHN2	ARHGEF3
AKAP9	TBC1D15	RIPPLY2	TMEM128
CREBZF	ZNF367	ZNF292	GULP1
CPEB3	NEUROG2	MAST4	MDM1
NR3C1	FOXO1	CARF	
YY1	DOCK1	ZFYVE16	
VEZF1	IER5	KIAA1109	
CNOT6L	SPOCK3	TMEM39A	
PTPRE	COL4A1	TMOD3	
TMEM132B	OPA1	ZNF518B	
ATAD5	GIGYF2	TVP23B	
RAB10	TOB1	ARHGAP12	
ICK	TLE1	KDM2B	
KIAA1217	VAPA	CNTN1	
MED14	ACTR3	ACVR1	
ITGA6	SYNGR3	PIAS1	
ARID4B	LRRC32	MORC3	
PLAG1	AKTIP	SPRY2	
PSD3	EFR3A	ACTL6A	
CRISPLD1	NR5A2	RALGDS	
CCDC126	MED6	TGFB3	
LRP6	NBEA	LMLN	
NR2F2	TCF3	RABGGTB	
ZNF800	AMMECR1	FAM105A	
POLR2K	FBXO34	UNC119B	
EDIL3	OLFML2B	SRSF11	
NFYB	USP34	ST6GALNAC3	
CASP8AP2	ZMYND8	CEPT1	
LRRC16A	HECTD1	SETX	

LET7D-3p

Gene short name
MEX3C

c

miRNA predicted targets (Score ≥95)

LET7A-5p Gene short name	LET7D-5p Gene short name	LET7F-5p Gene short name
SMARCAD1	SMARCAD1	SMARCAD1
FAM178A	FAM178A	FAM178A
LIN28B	LIN28B	LIN28B
GATM	LRIG3	ZBTB5
LRIG3	GATM	GNPTAB
GNPTAB	IGDCC3	GATM
BZW1	GNPTAB	LRIG3
ZNF322	ADAMTS8	ADRB2
ADAMTS8	C8orf58	BZW1
C8orf58	ZNF322	ADAMTS8
ADRB2	DNA2	PDP2
DNA2	ADRB2	DNA2
IGDCC3	BZW1	C8orf58
TTLL4	PARD6B	IGDCC3
NME6	TMPRSS2	TMPRSS2
TMPRSS2	NME6	NME6
HIC2	TTLL4	TTLL4
MAPK6	ZFYVE26	MAPK6
DMD	DMD	ZFYVE26
SCN4B	HIC2	SCN4B
ZFYVE26	MAPK6	HIC2
FZD3	SCN4B	DMD
LIMD2	TMEM2	COL3A1
SMIM3	FZD3	LIMD2
TMEM2	COL3A1	PCGF3
PCGF3	LIMD2	TMEM2
COL3A1	SMIM3	FZD3
ZBTB5	PCGF3	SMIM3
	ZBTB5	

LET7F-3p

Gene short name	Gene short name	Gene short name	Gene short name
RIMKLB	GNB4	NDC1	CBLN4
CDK19	ERF	PPP1R15B	DCAF6
FAM3C	TAOK1	SGTB	PDS5B
BTAF1	HNRNPD	CCNY	ASAH1
DAAM1	FYTTD1	SLC18A2	ANKRD44
SNAPC1	CRISPLD1	MARCH6	ADAMTSL3
CCNA2	LRP6	BTF3L4	ALDH1L2
DCUN1D4	POLR2K	B3GNT2	ARHGEF3
TSHZ3	EDIL3	F3	TMEM128
REV3L	CASP8AP2	ARRDC3	MDM1
AKAP9	LRRC16A	CCNE2	FAM105A
NR3C1	ARIH1	PTBP3	UNC119B
CNOT6L	HMGXB4	FBXO43	SRSF11
PTPRE	CAMTA1	BDNF	ST6GALNAC3
RAB10	TRPM7	GTF2A1	SETX
ICK	PAK2	SHOC2	SDC2
RICTOR	DCLK3	CMTM4	KLF4
SRSF2	WASF3	RDX	FZD6
EMP2	SYF2	PDZRN3	SLC23A2
PUM2	NASP	SGOL1	DLL4
FRS2	ZCCHC11	WDHD1	SOWAHC
SMARCA5	TBC1D15	DR1	EEA1
TMEM2	FOXO1	MBLAC2	DOPEY1
NUDT4	SPOCK3	ITM2B	GTF3C3
TRIM63	COL4A1	ZNF292	SPAST
PAN3	GIGYF2	MAST4	SP4
CLOCK	TOB1	CARF	GULP1
SMEK2	TLE1	ZFYVE16	
CREBZF	LRRC32	KIAA1109	
CPEB3	FBXO34	TMEM39A	
YY1	OLFML2B	ZNF518B	
VEZF1	ST5	KDM2B	
TMEM132B	ZNF367	CNTN1	
ATAD5	NEUROG2	PIAS1	
KIAA1217	DOCK1	ACTL6A	
MED14	IER5	LMLN	
ITGA6	OPA1	DLL1	
ARID4B	VAPA	LPHN2	
PLAG1	ACTR3	RIPPLY2	
PSD3	SYNGR3	TVP23B	
CCDC126	AKTIP	MALT1	
NR2F2	EFR3A	ARHGAP12	
ZNF800	NR5A2	MORC3	
NFYB	MED6	SPRY2	
ITCH	NBEA	RALGDS	
SORBS1	TCF3	TGFB3	
CEP76	AMMECR1	RABGGTB	
HMGCR	USP34	CEPT1	
ARHGAP20	ZMYND8	SH3GL3	
GLS	HECTD1	DCAF7	

Extended Data Table 2

(a) The list indicates the downregulated miRNAs in all three BCL7B-deficient cells compared to control Kato III cells. (b) The predicted targets (Score ≥ 95) of LET7A, D and F in each 3-prime site are shown. We used an online database, miRDB (http://www.mirdb.org/), for miRNA target prediction and functional annotations27. All of the predicted targets had target prediction scores ranging from 50 to 100. According to the description on the web site, a target with a prediction score > 80 is most likely to be a true candidate. The purple columns indicate the proteins expressed in the nucleus, and the reddish purple columns indicate the proteins expressed in the centrosome as indicated by the Human Protein Atlas database (http://www.proteinatlas.org/). (c) The predicted targets (score ≥ 95) of LET7A, D and F in each 5-prime site are shown. The target genes for the three miRNAs were highly shared. The purple columns indicate the proteins expressed in the nucleus.

Chr. No.	Ensemble gene_id	Gene short name	Δ1ratio a	Δ2ratio a	Δ3ratio a	Average
13q12.11	ENSG00000265710.1	AL161772.1	#DIV/0!	#DIV/0!	#DIV/0!	#DIV/0!
17q23.3	ENSG00000265695.1	MIR3064	#DIV/0!	#DIV/0!	#DIV/0!	#DIV/0!
2q33.1	ENSG00000212309.1	SNORD70	#DIV/0!	#DIV/0!	#DIV/0!	#DIV/0!
13q12.2	ENSG00000207500.1	SNORD102	#DIV/0!	#DIV/0!	#DIV/0!	#DIV/0!
10p11.22	ENSG00000222309.1	AL391839.1	4.75E+21	3.63E+21	9.28E+21	5.88E+21
11p15.5	ENSG00000130600.11	H19	1.265137	6.104297	131.805	46.39147
Xp22.31	ENSG00000205642.5	VCX3B	2.246958	55.87772	72.71897	43.61455
Xp22.31	ENSG00000182583.8	VCX	2.621333	50.82107	70.60833	41.35024
Xp22.31	ENSG00000169059.8	VCX3A	1.94196	45.27428	54.43711	33.88445
6p21.32	ENSG00000204248.6	COL11A2	2.067723	2.695578	51.20864	18.65731
4p15.32	ENSG00000137440.3	FGFBP1	3.217406	1.191111	34.26626	12.89159
11q12.1	ENSG00000254979.1	RP11-872D17.8	13.08093	7.282395	12.00345	10.78893
Xp21.2	ENSG00000099399.5	MAGEB2	18.50294	3.620105	8.613596	10.24555
11q14.2	ENSG00000123892.7	RAB38	2.785022	3.490802	16.28221	7.519344
17q21.2	ENSG00000128422.11	KRT17	2.835908	2.533559	13.7526	6.374021
11q23.3	ENSG00000076706.10	MCAM	1.292656	2.316508	11.88426	5.164475
7q21.3	ENSG00000105854.8	PON2	4.016785	2.879006	6.584437	4.493409
13q34	ENSG00000234603.2	AL356740.1	9.48664	1.947197	1.748528	4.394122
11q13.5	ENSG00000085741.8	WNT11	2.55257	1.635401	8.50128	4.22975
17q21.2	ENSG00000171401.10	KRT13	1.482846	1.224753	9.687596	4.131732
17q23.3	ENSG00000108622.6	ICAM2	2.23474	1.454792	8.058012	3.915848
11q14.2	ENSG00000109861.11	CTSC	2.598419	1.644403	6.726081	3.656301
16q13	ENSG00000205358.3	MT1H	1.015177	1.246137	8.648642	3.636652
17q21.2	ENSG00000108244.12	KRT23	3.387405	1.869976	5.512223	3.589868
6q21	ENSG00000203778.3	FAM229B	1.616234	5.707179	3.338826	3.55408
7p22.3	ENSG00000073067.9	CYP2W1	1.369095	1.786136	7.141407	3.432213
5q31.3	ENSG00000202111.1	VTRNA1-2	1.572397	5.651736	2.844856	3.35633
7p14.3	ENSG00000187258.9	NPSR1	1.070049	2.998446	5.939658	3.336051
17p13.2	ENSG00000091592.11	NLRP1	2.472387	1.720023	5.732178	3.308196
13q22.1	ENSG00000236972.1	FABP5P1	2.959277	1.187508	5.434948	3.193911
1p22.1	ENSG00000229567.1	RP4-717I23.2	5.028497	1.781128	2.71806	3.175895
17q25.3	ENSG00000185269.7	NOTUM	2.31948	1.378521	5.699806	3.132602
1q21.3	ENSG00000196154.7	S100A4	1.41061	2.820161	5.04377	3.091514
20q11.22	ENSG00000229230.2	MT1P3	2.532296	1.622456	5.063933	3.072895
16p11.2	ENSG00000213658.6	LAT	1.630795	2.766211	4.661137	3.019381
19q13.3	ENSG00000220988.1	SNORD88C	1.139611	1.775906	6.014489	2.976668
4q24	ENSG00000250920.1	RP11-297P16.4	2.244726	1.744972	4.891744	2.960481
4q13.3	ENSG00000169429.6	IL8	1.067833	3.674384	3.866658	2.869625
Xq28	ENSG00000198910.8	L1CAM	1.824732	2.337642	4.398519	2.853631
2p14	ENSG00000214533.3	KRT18P33	3.501213	1.009185	4.030202	2.846867
22q13.1	ENSG00000100097.7	LGALS1	1.81825	3.117829	3.594523	2.843534
14q22.2	ENSG00000125378.11	BMP4	1.122894	3.566194	3.838128	2.842405
3q13.31	ENSG00000243014.1	PTMAP8	4.118691	1.301792	2.919448	2.779977
10q26.2	ENSG00000180745.4	CLRN3	2.331945	1.439565	4.375691	2.715734
2q35	ENSG00000127824.9	TUBA4A	2.266863	1.169128	4.612242	2.682744
10q21.3	ENSG00000214881.4	TMEM14D	2.61436	2.454793	2.977083	2.682079
3p25.2	ENSG00000207496.1	SNORA7A	1.602659	2.422827	3.965843	2.663776
11q12.1	ENSG00000234964.3	FABP5P7	2.279835	1.226299	4.313548	2.606561
16q13	ENSG00000169715.10	MT1E	1.52059	1.422683	4.678538	2.540604
1q21.3	ENSG00000188643.6	S100A16	1.62165	1.738998	4.091994	2.484214

Chr. No.	Ensemble gene_id	Gene short name	Δ1ratio av	Δ2ratio av	Δ3ratio av	Average
2q11.2	ENSG00000212283.1	SNORD89	2.1730865	3.2275884	1.95977837	2.453484
2q32.3	ENSG00000266817.1	AC118063.1	3.38433	2.9117839	1.06079459	2.452303
2q25.1	ENSG00000213774.3	AC010904.1	2.7687177	1.2733432	3.28472036	2.44226
15q15.3	ENSG00000224677.1	AC011330.6	2.8674277	1.0364409	3.4104918	2.43812
15q25.2	ENSG00000197696.5	NMB	1.5382862	2.4634379	3.24681447	2.41618
21q22.3	ENSG00000226543.2	MYL6P1	2.7068527	1.433966	3.00302293	2.381281
8q21.13	ENSG00000164687.6	FABP5	1.4756903	1.1882296	4.39629114	2.353404
1q32.1	ENSG00000133067.13	LGR6	2.2150379	1.1887129	3.6329009	2.345551
9p22.3	ENSG00000214110.3	LDHAP4	1.8005171	1.7436961	3.49083467	2.345016
22q11.21	ENSG00000128228.4	SDF2L1	1.3271185	1.6057605	4.06871916	2.333866
Xq13.1	ENSG00000090776.5	EFNB1	2.6370446	1.2052149	3.12510239	2.322454
3p21.31	ENSG00000068001.9	HYAL2	1.3797293	1.1951226	4.38955685	2.32147
Yq11.222	ENSG00000185275.6	CD24P4	2.5941429	1.3090805	3.02617351	2.309799
1p36.33	ENSG00000188157.9	AGRN	1.8544066	1.1000179	3.96942953	2.307951
11p11.2	ENSG00000085117.7	CD82	1.1712213	1.3030087	4.41016194	2.294797
3p21.2	ENSG00000248487.4	ABHD14A	1.7564794	2.0182236	3.0977097	2.290804
12p13.1	ENSG00000207817.1	MIR614	2.0902043	1.4378046	3.29388161	2.273964
15q15.1	ENSG00000243789.6	JMJD7	3.9675418	1.0202993	1.80721025	2.265017
8q24.3	ENSG00000169427.2	KCNK9	1.6252556	2.0931824	3.07607038	2.264836
Xp11.3	ENSG00000069535.12	MAOB	2.1551261	1.8598606	2.74991702	2.254968
17q25.1	ENSG00000264624.1	MIR3615	3.2657625	1.3218801	2.17176473	2.253136
4q24	ENSG00000164039.10	BDH2	1.820414	1.91006	3.01290037	2.247791
21q22.3	ENSG00000160181.4	TFF2	1.0065702	3.5267315	2.20588924	2.246397
17q21.2	ENSG00000141696.8	LEPREL4	1.8536412	1.4416767	3.4408043	2.245374
6p21.33	ENSG00000213722.4	DDAH2	2.1784248	1.6766673	2.87040285	2.241832
17p13.3	ENSG00000167703.10	SLC43A2	2.4165214	1.1157631	3.19008167	2.240789
16q13	ENSG00000187193.8	MT1X	1.0787289	1.8388925	3.72781943	2.215147
8p21.3	ENSG00000120913.19	PDLIM2	2.4587172	1.4150774	2.75691561	2.210237
1p36.13	ENSG00000142632.12	ARHGEF19	1.8075008	1.0903389	3.71912801	2.205656
1p34.2	ENSG00000117385.11	LEPRE1	1.4402246	1.7524053	3.38364706	2.192092
16p13.3	ENSG00000161981.6	SNRNP25	1.1455975	1.0905625	4.30399158	2.180051
2q21.2	ENSG00000163046.11	ANKRD30BL	2.0399611	2.3484899	2.09164833	2.160033
17q21.31	ENSG00000204652.5	RPS26P8	2.2469378	1.9420845	2.20561805	2.131547
16p12.2	ENSG00000103356.11	EARS2	1.0197453	1.3507625	4.00920503	2.126571
Xp22.33 an	ENSG00000002586.13	CD99	1.588394	1.6448348	3.1320965	2.121775
2q37.3	ENSG00000063660.4	GPC1	1.6816904	1.089045	3.58770163	2.119479
22q11.1	ENSG00000100181.17	TPTEP1	1.0580983	2.163754	3.11997619	2.113943
Xq22.2	ENSG00000185222.7	WBP5	1.3558231	2.1527257	2.76250411	2.090351
11p15.4	ENSG00000129757.8	CDKN1C	1.0480511	1.6457395	3.55210397	2.081965
6q21	ENSG00000228834.1	RP11-249L21.4	1.9647717	2.2991286	1.96015949	2.074687
16q22.1	ENSG00000140939.10	NOL3	1.7783729	2.3016832	2.10078655	2.060281
20q13.13	ENSG00000231878.1	SNRPFP1	3.303913	1.0424125	1.82185855	2.056061
16p13.13	ENSG00000182108.5	DEXI	1.5892842	2.1650758	2.40377542	2.052712
19q13.2	ENSG00000104835.10	SARS2	2.0865769	1.2832989	2.76558965	2.045155
1q21.3	ENSG00000231416.1	RP11-422P24.9	2.3661757	1.8562151	1.90538485	2.042592
20q13.33	ENSG00000092758.11	COL9A3	1.4629909	1.8251771	2.82272187	2.036963
19q13.31	ENSG00000105767.2	CADM4	2.0103136	1.1679088	2.93084714	2.036357
18q21.33	ENSG00000206075.9	SERPINB5	1.3375099	2.7827293	1.98275188	2.03433
6p22.2	ENSG00000187990.4	HIST1H2BG	1.1851534	3.5813171	1.31854922	2.02834
2q32.1	ENSG00000231203.1	KRT8P10	3.0797369	1.1422344	1.85983239	2.027268
19q13.42	ENSG00000179820.11	MYADM	2.3427374	1.9639821	1.76052625	2.022415
19q13.12	ENSG00000089356.12	FXYD3	2.4935343	1.4966724	2.07194441	2.020717
22q11.23	ENSG00000240972.1	MIF	1.3561003	1.1312494	3.55421465	2.013855

Extended Data Table 3

The list of genes indicates the commonly upregulated mRNAs in all three BCL7B-deficient cell lines compared to control Kato III cells. Average ratios and chromosomal locations are shown.

a

Protein name in *C.elegans*	Function (Targets) in *C. elegans*	*C.elegans* mutant strain name	*Homo sapiens* ortholog protein	Function (Targets) in *Homo sapiens*
MET-2	Histone methyltransferase (H3K9me1/2)	met-2(ot861[met-2::mKate2])III	SETDB1	Histone methyltransferase (H3K9me3)
MES-4	Histone methyltransferase (H3K36me2/3)	nuc-119(ed3)III; stIs10325[mes-4::H1-wCherry+nuc-119(+)]	NSD1, NSD2, NSD3	Histone methyltransferase (NSD1: H3K36, H4K20) (NSD2: H3K27) (NSD3: H3K4, H3K27)
MES-2	Histone methyltransferase PRC2 complex subunit (H3K27)	mes-2(ax2059[mes-2::GFP])II	EZH2	Histone methyltransferase PRC2 complex subunit (H3K9, H3K27)
HPL-1	Binds HIS-24/H1K14me1 in vitro	hpl-1(ot841[hpl-2::mKate2])X	CBX3	Recognizes and binds methylated H3K9 tails.
HPL-2	Binds H3K9me1/2/3 in vitro	hpl-2(ot860[hpl-2::mKate2])III	CBX3?	Recognizes and binds methylated H3K9 tails.

b

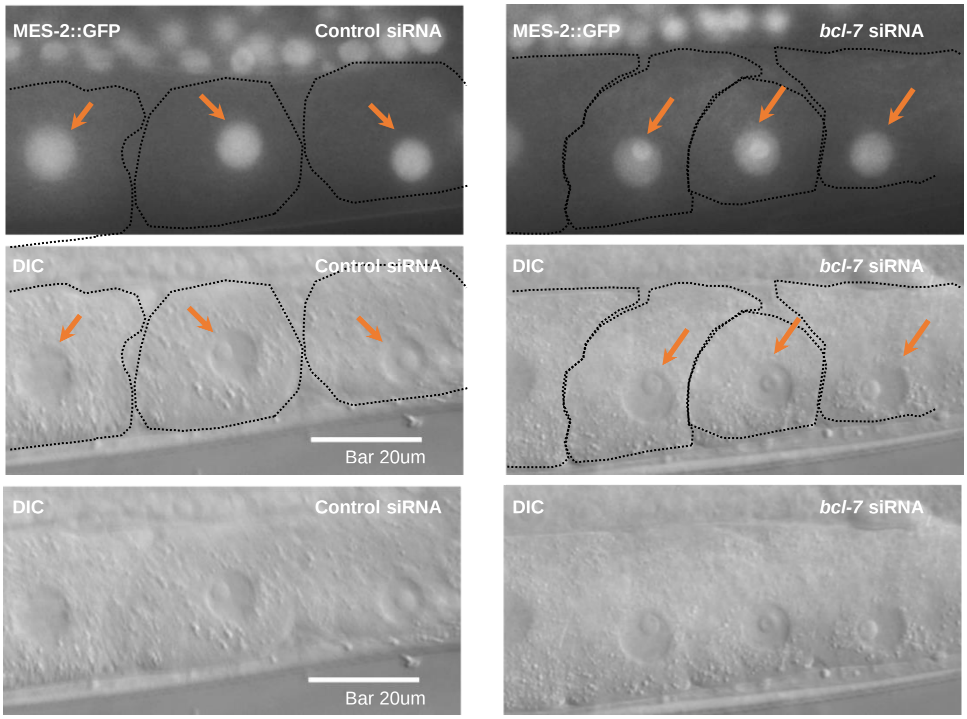

c

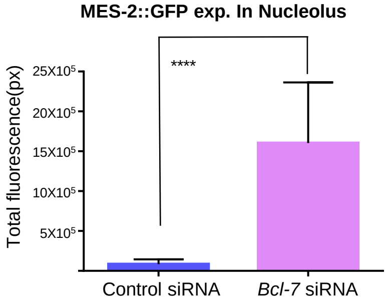

Extended Data Figure 5

Comparison between control siRNA and bcl-7 siRNA in several C. elegans mutants related to epigenetic markers. (a) List of C. elegans mutants. (b) Comparison between control siRNA and bcl-7 siRNA in the MES-2::GFP-carrying C. elegans mutant. Images of MES-2::GFP (top) and image showing the contrast between different levels of interference (middle and bottom) of oocyte cells. Arrows indicate nuclei (top and middle). Dotted lines indicate the edges of cells. (c) The total fluorescence (px) in the nucleolus was measured by ImageJ. Error bars, SEs (control siRNA, n=63, bcl-7 siRNA, n=63). The asterisk shows the statistical significance (****p<0.0001).

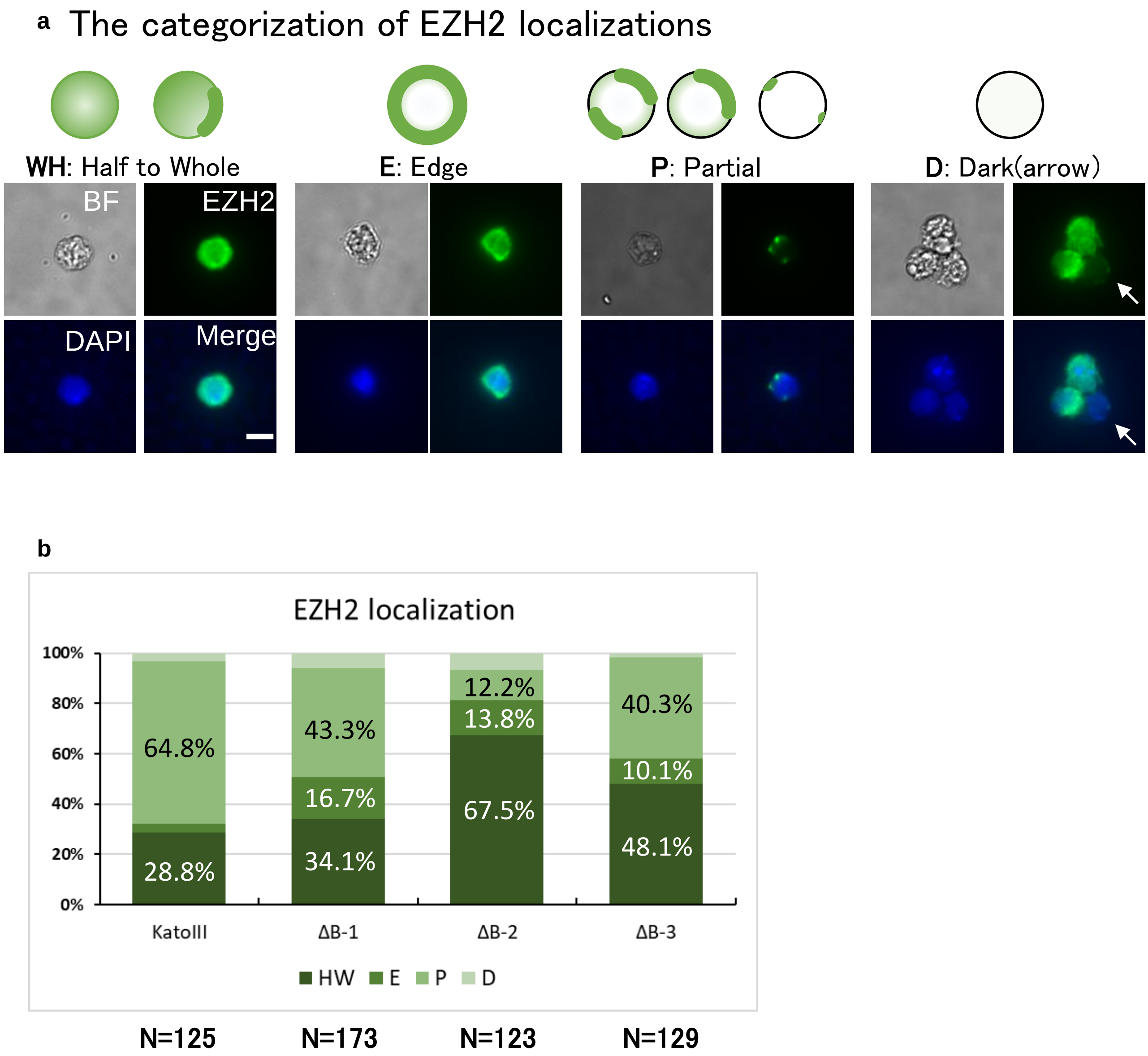

Extended Data Figure 6

The comparison of the EZH2 localization in KatoIII and BCL7B deficient cells by immunohistochemistry. (a) The categorization of EZH2 localization in KatoIII cells. The localizations of EZH2 were categorized into four types, Half to Whole (HW), Edge (E), Partial (P) and Dark (D). (b) The comparison of the EZH2 localization in KatoIII and BCL7B deficient cells. EZH2 localization tends to expand in BCL7B deficient cells.

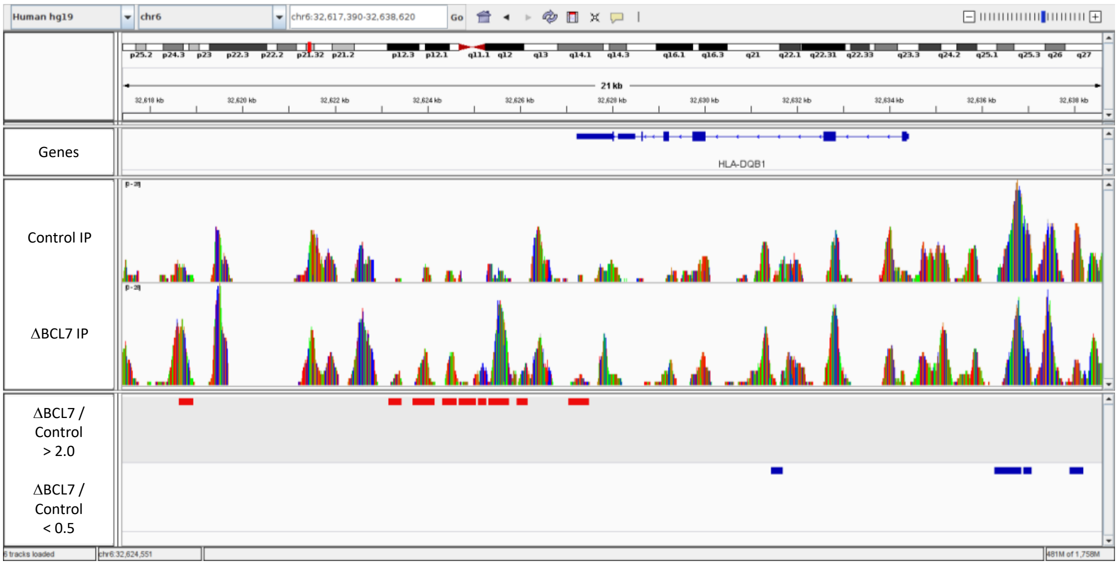

Extended Data Figure 7

An example of peak regions neighbouring the HLA-DQB1 gene as detected by ChIP-seq data analysis. For the details of the analysis, please refer to the Materials and Methods. Peak regions where the read depth of the sequenced data was derived from the ΔBCL7B cell line was greater than that derived from the parent cell line are indicated by red boxes in the lower panel. In contrast, regions where the read depth of the sequenced data derived from the ΔBCL7B cell line was lower than that derived from the parent cell line are indicated by blue boxes in the lower panel.

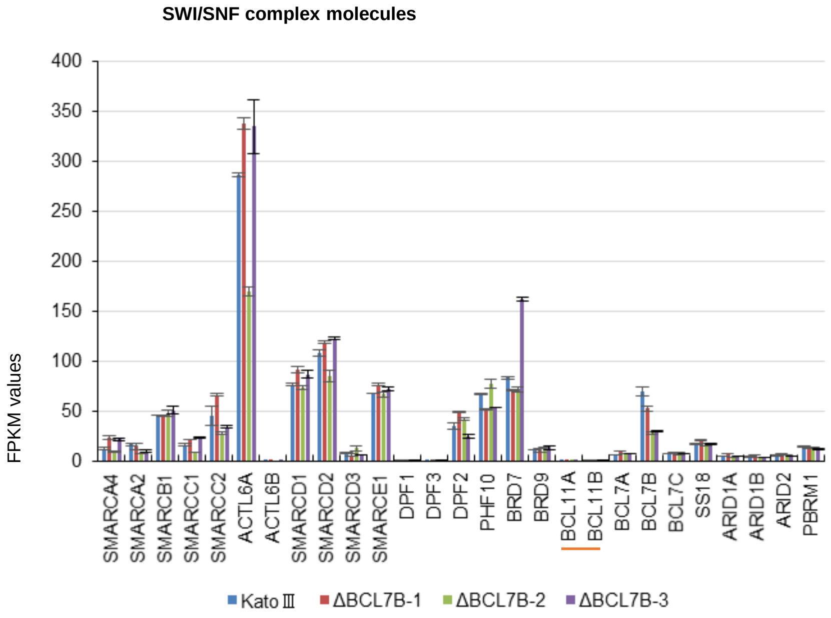

Extended Data Figure 8

(a) The FPKM values of SWI/SNF complex molecules in the RNA-seq data obtained from the Kato III and ΔBCL7B cell lines. The BCL11A/BCL11B with red underlining were not detected.

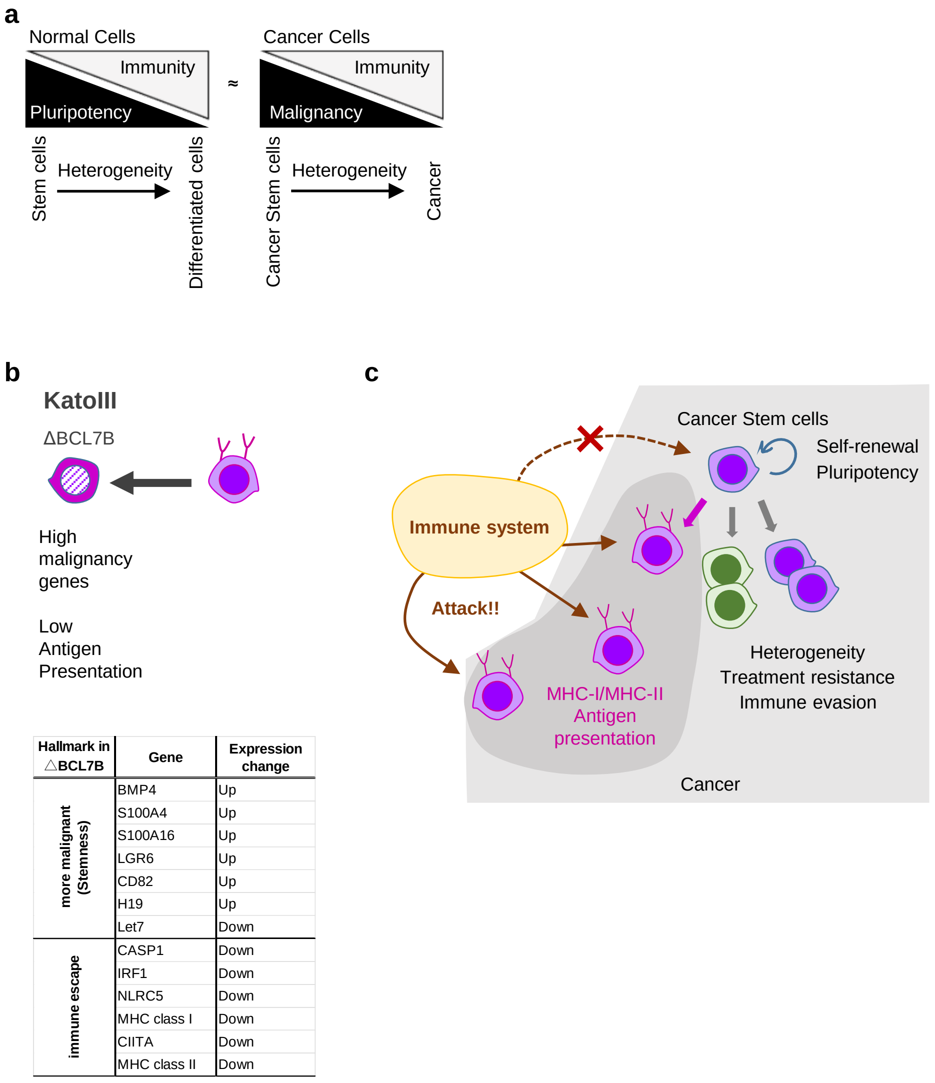

Hallmark in △BCL7B	Gene	Expression change
more malignant (Stemness)	BMP4	Up
	S100A4	Up
	S100A16	Up
	LGR6	Up
	CD82	Up
	H19	Up
	Let7	Down
immune escape	CASP1	Down
	IRF1	Down
	NLRC5	Down
	MHC class I	Down
	CIITA	Down
	MHC class II	Down

Extended Data Figure 9

The summary diagram in this study and the relation of the findings to previously obtained knowledge. (a) A simplified concept of the relationship between immunity and the regeneration potential of organisms, immunity and the pluripotency potential of cells, and immunity and the pluripotency potential of cancer cells. (b) Summary of the characteristics of BCL7B-deficient Kato III cells. The cells become more malignant when antigen presentation is low. These properties are similar to those of cancer stem cells. (c) The summary diagram of this study combined with conventional knowledge. BCL7B expression is very important for antigen presentation, and it is key to the immune surveillance system.
