# Supplementary material for: BCL7B, a SWI/SNF complex subunit, orchestrates cancer immunity and stemness
Source: BMC Cancer. 2023 Aug 30;23:811. doi: 10.1186/s12885-023-11321-3 (PMC10466690; doi:10.1186/s12885-023-11321-3)
Supplement: Supplementary file 2 — Additional file 2. [file 12885_2023_11321_MOESM2_ESM.zip › Supplymentary Table 1 References.docx]

The References of Supplementary Table 1

Chr.1p

Li, Y. *et al*. ARHGEF19 interacts with BRAF to activate MAPK signaling during the tumorigenesis of non-small cell lung cancer. Int J Cancer. **142**, 7, 1379-1391. (2018)

Li, X. *et al*. Oncogenic Properties of NEAT1 in Prostate Cancer Cells Depend on the CDC5L-AGRN Transcriptional Regulation Circuit. Cancer Res. **78**, 15, 4138-4149. (2018)

Chr.1q

Cortesi, E. & Ventura, J.J. Lgr6: From Stemness to Cancer Progression. J Lung Health Dis. **3**, 1, 12-15. (2019)

Nirala, B., Baskin, D. & Yun, K. Cell-autonomous and non-autonomous functions of S100A4 in regulating stemness, mesenchymal transition, and metastasis. Oncoscience. **4**, 11-12, 166-167. (2017)

Tomiyama, N. *et al*. S100A16 up-regulates Oct4 and Nanog expression in cancer stem-like cells of Yumoto human cervical carcinoma cells. Oncol Lett. **15**, 6, 9929-9933. (2018)

Chr.2q

Krishnan, P. *et al*. Profiling of Small Nucleolar RNAs by Next Generation Sequencing: Potential New Players for Breast Cancer Prognosis. PLoS One. **11**, 9, e0162622. (2016)

Garaud, S. *et al*. Antigen Specificity and Clinical Significance of IgG and IgA Autoantibodies Produced in situ by Tumor-Infiltrating B Cells in Breast Cancer. Front Immunol. **9**, 2660. (2018)

Luo, H., Zhao, X., Wan, X., Huang, S. & Wu, D. Gene microarray analysis of the lncRNA expression profile in human urothelial carcinoma of the bladder. Int J Clin Exp Med. **7**, 5, 1244-54. (2014)

Strassel, C. *et al*. An essential role for α4A-tubulin in platelet biogenesis. Life Sci Alliance. **2**, 1, e201900309. (2019)

Melo, S.A. *et al*. Glypican-1 identifies cancer exosomes and detects early pancreatic cancer.

Nature. **523**, 7559, 177-82. (2015)

Li, J. *et al*. GPC1 exosome and its regulatory miRNAs are specific markers for the detection and target therapy of colorectal cancer. J Cell Mol Med. **21**, 5, 838-847. (2017)

Li, J. *et al*. The clinical significance of circulating GPC1 positive exosomes and its regulative miRNAs in colon cancer patients. Oncotarget. **8**, 60, 101189-101202. (2017)

Chr.3p

Savci-Heijink, C.D., Halfwerk, H., Koster, J., Horlings, H.M. & van de Vijver, M.J. A specific gene expression signature for visceral organ metastasis in breast cancer. BMC Cancer. **19**, 1, 333. (2019)

Maierthaler, M. *et al*. S100P and HYAL2 as prognostic markers for patients with triple-negative breast cancer. Exp Mol Pathol. **99**, 1, 180-7. (2015)

Jin, Z. *et al*. The suppressive role of HYAL1 and HYAL2 in the metastasis of colorectal cancer.J Gastroenterol Hepatol. [Epub ahead of print] (2019)

Zhang, Y. *et al*. H/ACA Box Small Nucleolar RNA 7A Promotes the Self-Renewal of Human Umbilical Cord Mesenchymal Stem Cells. Stem Cells. **35**, 1, 222-235. (2017)

Chr. 4p

Huang, W. *et al*. Sox12, a direct target of FoxQ1, promotes hepatocellular carcinoma metastasis through up-regulating Twist1 and FGFBP1. Hepatology. **61**, 6, 1920-33. (2015)

Chr. 4q

Fortner, R.T., Damms-Machado, A. & Kaaks, R. Systematic review: Tumor-associated antigen autoantibodies and ovarian cancer early detection. Gynecol Oncol. **147**, 2, 465-480. (2017)

Yang, W.C. *et al*. Human BDH2, an anti-apoptosis factor, is a novel poor prognostic factor for de novo cytogenetically normal acute myeloid leukemia. J Biomed Sci. **20**, 58. (2013)

Cui, X.P. *et al*. LncRNA TP73-AS1 sponges miR-141-3p to promote the migration and invasion of pancreatic cancer cells through the up-regulation of BDH2. Biosci Rep. **39**, 3, BSR20181937. (2019)

Chr.5p

Ashida, S., Kawada, C. & Inoue, K. Stromal regulation of prostate cancer cell growth by mevalonate pathway enzymes HMGCS1 and HMGCR. Oncol Lett. **14**, 6, 6533-6542. (2017)

Fatima, S. *et al*. Transforming capacity of two novel genes JS-1 and JS-2 located in chromosome 5p and their overexpression in human esophageal squamous cell carcinoma. Int J Mol Med. **17**, 1, 159-70. (2006)

Chr. 5q

Helbo, A.S. *et al*. Hypermethylation of the VTRNA1-3 Promoter is Associated with Poor Outcome in Lower Risk Myelodysplastic Syndrome Patients. Genes (Basel). **6**, 4, 977-90. (2015)

Chr. 6p

Mengual, L. *et al*. Using gene expression from urine sediment to diagnose prostate cancer: development of a new multiplex mRNA urine test and validation of current biomarkers. BMC Cancer. **16**, 76. (2016)

Yang, Y. *et al*. High HSF4 expression is an independent indicator of poor overall survival and recurrence free survival in patients with primary colorectal cancer. IUBMB Life. **69**, 12, 956-961. (2017)

Khor, GH. *et al*. DNA methylation profiling revealed promoter hypermethylation-induced silencing of p16, DDAH2 and DUSP1 in primary oral squamous cell carcinoma. Int J Med Sci. **10**, 12, 1727-39. (2013)

Chr. 7p

Pulkkinen, V. *et al*. Neuropeptide S receptor 1 (NPSR1) activates cancer-related pathways and is widely expressed in neuroendocrine tumors. Virchows Arch. **465**, 2, 173-83. (2014)

Sarvestani, S.K. *et al*. Cancer-predicting transcriptomic and epigenetic signatures revealed for ulcerative colitis in patient-derived epithelial organoids. Oncotarget. **9**, 47, 28717-28730. (2018)

Chung, F.F., Mai, C.W., Ng, P.Y. & Leong, C.O. Cytochrome P450 2W1 (CYP2W1) in Colorectal Cancers. Curr Cancer Drug Targets. **16**, 1, 71-8. (2016)

Chr. 7q

Krüger, M., Pabst, A.M., Al-Nawas, B., Horke, S. & Moergel, M. Paraoxonase-2 (PON2) protects oral squamous cell cancer cells against irradiation-induced apoptosis. J Cancer Res Clin Oncol. **141**, 10, 1757-66. (2015)

Nagarajan, A. *et al*. Paraoxonase 2 Facilitates Pancreatic Cancer Growth and Metastasis by Stimulating GLUT1-Mediated Glucose Transport. Mol Cell. **67**, 4, 685-701.e6. (2017)

Bacchetti, T., Ferretti, G. & Sahebkar, A. The role of paraoxonase in cancer. Semin Cancer Biol. **56**, 72-86. (2019)

Chr. 8p

Bowe, R.A. *et al*. PDLIM2 regulates transcription factor activity in epithelial-to-mesenchymal transition via the COP9 signalosome. Mol Biol Cell. **25**, 1, 184-95. (2014)

Cox, O.T. *et al*. PDLIM2 Is a Marker of Adhesion and β-Catenin Activity in Triple-Negative Breast Cancer. Cancer Res. **79**, 10, 2619-2633. (2019)

Chr. 8q

Kawaguchi, K. *et al*. The cancer-promoting gene fatty acid-binding protein 5 (FABP5) is epigenetically regulated during human prostate carcinogenesis. Biochem J. **473**, 4, 449-61. (2016)

Wang, W. *et al*. FABP5 correlates with poor prognosis and promotes tumor cell growth and metastasis in cervical cancer. Tumour Biol. **37**, 11, 14873-14883. (2016)

Sun, H. *et al*. A monoclonal antibody against KCNK9 K(+) channel extracellular domain inhibits tumour growth and metastasis. Nat Commun. **7**, 10339. (2016)

Chr. 10q

Mallanna, S.K., Cayo, M.A., Twaroski, K., Gundry, R.L., & Duncan, S.A. Mapping the Cell-Surface N-Glycoproteome of Human Hepatocytes Reveals Markers for Selecting a Homogeneous Population of iPSC-Derived Hepatocytes. Stem Cell Reports. **7**, 3, 543-556. (2016)

Chr. 11p

Ji, H. *et al*. CD82 supports survival of childhood acute myeloid leukemia cells via activation of Wnt/β-catenin signaling pathway. Pediatr Res. **85**, 7, 1024-1031. (2019)

Stampone, E. *et al*. Genetic and Epigenetic Control of CDKN1C Expression: Importance in Cell Commitment and Differentiation, Tissue Homeostasis and Human Diseases. Int J Mol Sci. **19**, 4, E1055. Review (2018)

Chen, S. *et al*. LncRNAs and their role in cancer stem cells. Oncotarget. **8**, 66, 110685-110692. Review (2017)

Sasaki, N. *et al*. H19 long non-coding RNA contributes to sphere formation and invasion through regulation of CD24 and integrin expression in pancreatic cancer cells. Oncotarget. **9**, 78, 34719-34734. (2018)

Chr. 11q

Tian, S. *et al*. Secreted AGR2 promotes invasion of colorectal cancer cells via Wnt11-mediated non-canonical Wnt signaling. Exp Cell Res. **364**, 2, 198-207. (2018)

Zhang, G.P., Yue, X. & Li, S.Q. Cathepsin C Interacts with TNF-α/p38 MAPK Signaling Pathway to Promote Proliferation and Metastasis in Hepatocellular Carcinoma. Cancer Res Treat. (2019)

Li, B.Y., He, L.J., Zhang, X.L., Liu, H. & Liu, B. High expression of RAB38 promotes malignant progression of pancreatic cancer. Mol Med Rep. **19**, 2, 909-918. (2019)

Tripathi, S.C. *et al*. MCAM Mediates Chemoresistance in Small-Cell Lung Cancer via the PI3K/AKT/SOX2 Signaling Pathway. Cancer Res. **77**, 16, 4414-4425. (2017)

Chr. 12p

Zhang, J., Gao, D. & Zhang, H. Upregulation of miR-614 promotes proliferation and inhibits apoptosis in ovarian cancer by suppressing PPP2R2A expression. Mol Med Rep. **17**, 5, 6285-6292. (2018)

Chr. 14q

Catalano, V. *et al*. Activated Thyroid Hormone Promotes Differentiation and Chemotherapeutic Sensitization of Colorectal Cancer Stem Cells by Regulating Wnt and BMP4 Signaling. Cancer Res. **76**, 5, 1237-44. (2016)

Yokoyama, Y. *et al*. Autocrine BMP-4 Signaling Is a Therapeutic Target in Colorectal Cancer.

Cancer Res. **77**, 15, 4026-4038. (2017)

Chr. 15q

Chen, C. *et al*. Glycoprotein nmb Is Exposed on the Surface of Dormant Breast Cancer Cells and Induces Stem Cell-like Properties. Cancer Res. **78**, 22, 6424-6435. (2018)

Xie, R. *et al*. Role of the kringle-like domain in glycoprotein NMB for its tumorigenic potential.

Cancer Sci. **110**, 7, 2237-2246. (2019)

Liu, H. *et al*. Clipping of arginine-methylated histone tails by JMJD5 and JMJD7. Proc Natl Acad Sci U S A. **114**, 37, E7717-E7726. (2017)

Chr. 16p

Sato, K., Miyamoto, M., Takano, M., Furuya, K. & Tsuda, H. Significant relationship between the LAT1 expression pattern and chemoresistance in ovarian clear cell carcinoma. Virchows Arch. **474**, 6, 701-710. (2019)

Chr. 16q

Zheng, Y. *et al*. Metallothionein 1H (MT1H) functions as a tumor suppressor in hepatocellular carcinoma through regulating Wnt/β-catenin signaling pathway. BMC Cancer. **17**, 1, 161. (2017)

Demidenko, R. *et al*. Decreased expression of MT1E is a potential biomarker of prostate cancer progression. Oncotarget. **8**, 37, 61709-61718. (2017)

Liu, Z. *et al*. Metallothionein 1 family profiling identifies MT1X as a tumor suppressor involved in the progression and metastastatic capacity of hepatocellular carcinoma. Mol Carcinog. **57**, 11, 1435-1444. (2018)

Stanley, R.F. *et al*. A myeloid tumor suppressor role for NOL3. J Exp Med. **214**, 3, 753-771. (2017)

Chr. 17p

Wei, Y. *et al*. NLRP1 Overexpression Is Correlated with the Tumorigenesis and Proliferation of Human Breast Tumor. Biomed Res Int. 4938473. (2017)

Wang, Q. & Holst, J. L-type amino acid transport and cancer: targeting the mTORC1 pathway to inhibit neoplasia. Am J Cancer Res. **5**, 4, 1281-94. Review (2015)

Chr. 17q

Birkenkamp-Demtröder, K. *et al*. Keratin23 (KRT23) knockdown decreases proliferation and affects the DNA damage response of colon cancer cells. PLoS One. **8**, 9, e73593. (2013)

Hu, W.Y. *et al*. Isolation and functional interrogation of adult human prostate epithelial stem cells at single cell resolution. Stem Cell Res. **23**, 1-12. (2017)

Xu, H. *et al*. CD44 correlates with clinicopathological characteristics and is upregulated by EGFR in breast cancer. Int J Oncol. **49**, 4, 1343-50. (2016)

Wan, B., Zeng, Q., Tang, X.Z. & Tang, Y.X. P3H4 affects renal carcinoma through up-regulating miR-1/133a. Eur Rev Med Pharmacol Sci. **22**, 16, 5180-5186. (2018)

Li, W., Ye, L., Chen, Y. & Chen, P. P3H4 is correlated with clinicopathological features and prognosis in bladder cancer. World J Surg Oncol. **16**, 1, 206. (2018)

Bai, L., Wang, H., Wang, A.H., Zhang, L.Y. & Bai, J. MicroRNA-532 and microRNA-3064 inhibit cell proliferation and invasion by acting as direct regulators of human telomerase reverse transcriptase in ovarian cancer. PLoS One. **12**, 3, e0173912. (2017)

Wang, C., Liao, H., Sun, H., Zhang, Y. & Cao, Z. MicroRNA-3064-3p regulates the differentiation of cementoblasts through targeting DKK1. J Periodontal Res. **53**, 5, 705-713. (2018)

Kim, S.T. *et al*. Transcriptome analysis of CD133-positive stem cells and prognostic value of survivin in colorectal cancer. Cancer Genomics Proteomics. **11**, 5, 259-66. (2014)

Yoon, J.H. *et al*. NOTUM Is Involved in the Progression of Colorectal Cancer. Cancer Genomics Proteomics. **15**, 6, 485-497. (2018)

Chr.18q

Chang, I.W. *et al*. SERPINB5 Expression: Association with CCRT Response and Prognostic Value in Rectal Cancer. Int J Med Sci. **15**, 4, 376-384. (2018)

Chr.19q

Xue, Y. *et al*. SOX9/FXYD3/Src Axis Is Critical for ER+ Breast Cancer Stem Cell Function.

Mol Cancer Res. **17**, 1, 238-249. (2019)

Rivera, H. *et al*. A new mutation in the gene encoding mitochondrial seryl-tRNA synthetase as a cause of HUPRA syndrome. BMC Nephrol. **14**, 195. (2013)

Scott, M.S. *et al*. Human box C/D snoRNA processing conservation across multiple cell types.

Nucleic Acids Res. **40**, 8, 3676-88. (2012)

Saito, M. *et al*. Decreased expression of CADM1 and CADM4 are associated with advanced stage breast cancer. Oncol Lett. **15**, 2, 2401-2406. (2018)

Wang, Q. *et al*. Membrane protein hMYADM preferentially expressed in myeloid cells is up-regulated during differentiation of stem cells and myeloid leukemia cells. Life Sci. **80**, 5, 420-9. (2007)

Chr. 20q

Zhou, M., Gao, M., Luo, Y., Gui, R. & Ji, H. Long non-coding RNA metallothionein 1 pseudogene 3 promotes p2y12 expression by sponging miR-126 to activate platelet in diabetic animal model.

Platelets. **30**, 4, 452-459. (2019)

Lv X. *et al*. Identification of potential key genes and pathways predicting pathogenesis and prognosis for triple-negative breast cancer. Cancer Cell Int. **19**, 172. (2019)

Chr.21q

Dubeykovskaya, Z. *et al*. Neural innervation stimulates splenic TFF2 to arrest myeloid cell expansion and cancer. Nat Commun. **7**, 10517. (2016)

Ishibashi, Y. *et al*. Serum TFF1 and TFF3 but not TFF2 are higher in women with breast cancer than in women without breast cancer. Sci Rep. **7**, 1, 4846. (2017)

Chr.22q

Ruvolo, P.P. Galectins as regulators of cell survival in the leukemia niche. Adv Biol Regul. **71**, 41-54. (2019)

Zhu, J. *et al*. Galectin-1 induces metastasis and epithelial-mesenchymal transition (EMT) in human ovarian cancer cells via activation of the MAPK JNK/p38 signalling pathway. Am J Transl Res. **11**, 6, 3862-3878. (2019)

Ding, H., Liu, J., Zou, R., Cheng, P. & Su, Y. Long non-coding RNA TPTEP1 inhibits hepatocellular carcinoma progression by suppressing STAT3 phosphorylation. J Exp Clin Cancer Res. **38**, 1, 189. (2019)

Willis, S. *et al*. Single Gene Prognostic Biomarkers in Ovarian Cancer: A Meta-Analysis. PLoS One. **11**, 2, e0149183. (2016)

O'Reilly, C., Doroudian, M., Mawhinney, L. & Donnelly, S.C. Targeting MIF in Cancer: Therapeutic Strategies, Current Developments, and Future Opportunities. Med Res Rev. **36**, 3, 440-60. (2016)

Yang, S. *et al*. A Novel MIF Signaling Pathway Drives the Malignant Character of Pancreatic Cancer by Targeting NR3C2. Cancer Res. **76**, 13, 3838-50. (2016)

Chr.Xp

Sharpe, M.A. & Baskin, D.S. Monoamine oxidase B levels are highly expressed in human gliomas and are correlated with the expression of HiF-1α and with transcription factors Sp1 and Sp3. Oncotarget. **7**, 3, 3379-93. (2016)

Yin, L., Li, J., Liao, C.P. & Jason, Wu B. Monoamine Oxidase Deficiency Causes Prostate Atrophy and Reduces Prostate Progenitor Cell Activity. Stem Cells. **36**, 8, 1249-1258. (2018)

Peche, L.Y. *et al*. Human MageB2 Protein Expression Enhances E2F Transcriptional Activity, Cell Proliferation, and Resistance to Ribotoxic Stress. J Biol Chem. **290**, 49, 29652-62. (2015)

Jin, S. *et al*. Cancer/testis antigens (CTAs) expression in resected lung cancer. Onco Targets Ther. **11**, 4491-4499. (2018)

Taguchi, A. *et al*. A search for novel cancer/testis antigens in lung cancer identifies VCX/Y genes, expanding the repertoire of potential immunotherapeutic targets. Cancer Res. **74**, 17, 4694-705. (2014)

Deng, H. *et al*. Histone H3.3K27M Mobilizes Multiple Cancer/Testis (CT) Antigens in Pediatric Glioma. Mol Cancer Res. **16**, 4, 623-633. (2018)

Chung, S.S. *et al*. CD99 is a therapeutic target on disease stem cells in myeloid malignancies.

Sci Transl Med. **9**, 374, eaaj2025. (2017)

Chr. Xq

Arthur, A., Nguyen, T.M., Paton, S., Zannettino, A.C.W. & Gronthos, S. Loss of EfnB1 in the osteogenic lineage compromises their capacity to support hematopoietic stem/progenitor cell maintenance. Exp Hematol. **69**, 43-53. (2019)

Tang, R. *et al*. WW domain binding protein 5 induces multidrug resistance of small cell lung cancer under the regulation of miR-335 through the Hippo pathway. Br J Cancer. **115**, 2, 243-51. (2016)

Bao, S. *et al*. Targeting cancer stem cells through L1CAM suppresses glioma growth. Cancer Res. **68**, 15, 6043-8. (2008)

Altevogt, P., Doberstein, K. & Fogel, M. L1CAM in human cancer. Int J Cancer. **138**, 7, 1565-76. (2016)
